# Control of nongenetic heterogeneity in growth rate and stress tolerance of *Saccharomyces cerevisiae* by cyclic AMP-regulated transcription factors

**DOI:** 10.1371/journal.pgen.1007744

**Published:** 2018-11-02

**Authors:** Shuang Li, Daniella M. Giardina, Mark L. Siegal

**Affiliations:** Center for Genomics and Systems Biology, Department of Biology, New York University, New York, New York, United States of America; Pacific Northwest Research Institute, UNITED STATES

## Abstract

Genetically identical cells exhibit extensive phenotypic variation even under constant and benign conditions. This so-called nongenetic heterogeneity has important clinical implications: within tumors and microbial infections, cells show nongenetic heterogeneity in growth rate and in susceptibility to drugs or stress. The budding yeast, *Saccharomyces cerevisiae*, shows a similar form of nongenetic heterogeneity in which growth rate correlates positively with susceptibility to acute heat stress at the single-cell level. Using genetic and chemical perturbations, combined with high-throughput single-cell assays of yeast growth and gene expression, we show here that heterogeneity in intracellular cyclic AMP (cAMP) levels acting through the conserved Ras/cAMP/protein kinase A (PKA) pathway and its target transcription factors, Msn2 and Msn4, underlies this nongenetic heterogeneity. Lower levels of cAMP correspond to slower growth, as shown by direct comparison of cAMP concentration in subpopulations enriched for slower vs. faster growing cells. Concordantly, an endogenous reporter of this pathway’s activity correlates with growth in individual cells. The paralogs Msn2 and Msn4 differ in their roles in nongenetic heterogeneity in a way that demonstrates slow growth and stress tolerance are not inevitably linked. Heterogeneity in growth rate requires each, whereas only Msn2 is required for heterogeneity in expression of Tsl1, a subunit of trehalose synthase that contributes to acute-stress tolerance. Perturbing nongenetic heterogeneity by mutating genes in this pathway, or by culturing wild-type cells with the cell-permeable cAMP analog 8-bromo-cAMP or the PKA inhibitor H89, significantly impacts survival of acute heat stress. Perturbations that increase intracellular cAMP levels reduce the slower-growing subpopulation and increase susceptibility to acute heat stress, whereas PKA inhibition slows growth and decreases susceptibility to acute heat stress. Loss of Msn2 reduces, but does not completely eliminate, the correlation in individual cells between growth rate and acute-stress survival, suggesting a major role for the Msn2 pathway in nongenetic heterogeneity but also a residual benefit of slow growth. Our results shed light on the genetic control of nongenetic heterogeneity and suggest a possible means of defeating bet-hedging pathogens or tumor cells by making them more uniformly susceptible to treatment.

## Author summary

Nongenetic heterogeneity exists when a trait differs among individuals that have identical genotypes and environments. A clonal population can maximize its long-term success in an uncertain environment by diversifying its phenotypes via nongenetic heterogeneity: the currently unfavored ones may become the favored ones when conditions change. Nongenetic heterogeneity has clinical relevance. For example, populations of tumor cells or infectious microbes show cell-to-cell differences in growth and in drug or stress tolerance. This heterogeneity hampers efficient treatment and can potentiate harmful evolution of a tumor or pathogen. We show that in budding yeast, heterogeneity in intracellular cyclic AMP levels acting through the conserved Ras/cAMP/protein kinase A (PKA) pathway and its target transcription factors, Msn2 and Msn4, underlies the nongenetic heterogeneity of both single-cell growth rate and acute heat-stress tolerance. Perturbations of this pathway significantly affect population survival upon acute heat stress. These results illuminate a mechanism of nongenetic heterogeneity and suggest the potential value of antitumor or antifungal treatment strategies that target nongenetic heterogeneity to render the tumor or pathogen population more uniformly susceptible to a second drug that aims to kill.

## Introduction

For any particular trait, genetically identical individuals raised in identical environments may still differ. This phenomenon is termed phenotypic variability or nongenetic heterogeneity [1]. Examples of nongenetic heterogeneity include clonal microbial cultures in which cells differ in gene activity, growth and tolerance of stress or drug treatments [2–8]. Tumor cells also display nongenetic heterogeneity in gene expression, growth and chemotherapy tolerance [9–14]. Nongenetic heterogeneity of traits important for growth and survival would reduce fitness in a constant environment but can provide an evolutionary advantage when conditions fluctuate unpredictably, by enabling a clonal population to hedge its bets [3, 4, 14–16] or to explore and persist in unfamiliar niches as a precursor to genetic adaptation [11, 13, 17, 18]. In general, the molecular mechanisms that generate nongenetic heterogeneity are poorly understood [1]. Elucidating these mechanisms is an important goal because it can advance our understanding of how populations—including populations of pathogens or cancer cells—adapt to complex, changing environments.

We previously described a putative bet-hedging system in the budding yeast, *Saccharomyces cerevisiae* [3]. *S*. *cerevisiae* is not only a powerful model organism that has yielded key insights into cancer [19–21] and the evolution of clonal lineages [22–24], but is also an opportunistic pathogen [25–28]. Time-lapse microscopy of microcolonies founded by individual yeast cells revealed that genetically identical cells grown in the same, benign conditions have a wide range of cell-division rates and differ in their tolerance of acute heat stress [3]. Here we use the term “acute” to emphasize that the stress is sufficiently strong and sudden as to require cells to be prepared in advance rather than to mount a stress response to survive. Growth and acute stress tolerance are negatively correlated: slower-growing cells, which are at a disadvantage under benign conditions, disproportionally survive acute heat stress [3]. Slow growth and high stress tolerance also correlate at the single-cell level with high expression of the protein Tsl1, a component of the trehalose-synthase complex [3]. Stress induces production of Tsl1 and of trehalose, which preserves protein folding and acts as a storage carbohydrate [29, 30]. Under benign conditions, most cells express very little Tsl1, but some express much more [3]. Synthesis of trehalose in the latter cells could prepare them to withstand acute stress, and indeed a *tsl1*-deleted strain survives acute heat stress poorly compared with an isogenic wild-type strain [3]. Although a connection between slow growth and stress tolerance has been observed in several systems [4, 14, 31, 32], the two are not inextricably linked, as the *tsl1* deletion does not appreciably alter the distribution of microcolony growth rates [3].

The variability of microcolony growth rates differs between natural isolates of budding yeast [6, 33], implying both that there is a genetic basis of differences in nongenetic heterogeneity and that it is likely an ecologically relevant phenomenon. We therefore sought to elucidate the molecular mechanism that generates nongenetic heterogeneity in yeast-cell growth and acute stress tolerance. Our starting point was the regulation of *TSL1*. We reasoned that a regulatory pathway controlling cell-to-cell differences in *TSL1* expression would be an excellent candidate for coordinately controlling the nongenetic heterogeneity of growth and acute stress tolerance.

**Fig 1:**
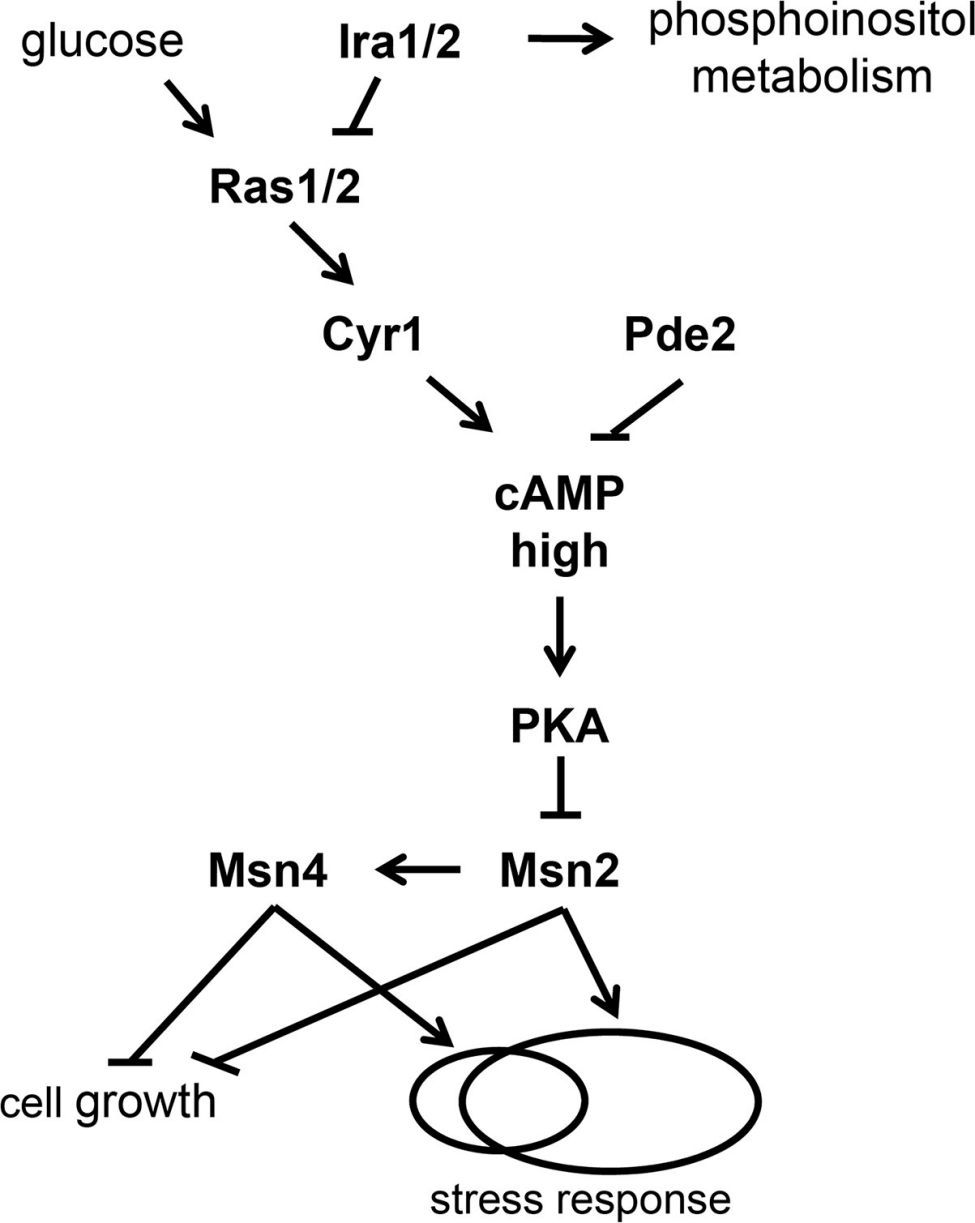
Standard model of the Ras/cAMP/PKA/Msn2/4 pathway. Under benign, glucose-replete conditions, the GTPases Ras1 and Ras2 exist predominantly in their GTP-bound form, which stimulates activity of the adenylyl cyclase Cyr1, thereby increasing intracellular cAMP concentration. Under glucose starvation, intracellular cAMP concentration decreases by virtue of the countervailing effects of the GTPase-activating proteins Ira1 and Ira2, which cause Ras1 and Ras2 to exist predominantly in their GDP-bound form, and the phosphodiesterases Pde1 and Pde2, which convert cAMP to AMP. Ira1 and Ira2 also positively regulate phosphoinositol metabolism independent of the Ras/cAMP pathway. cAMP activates PKA by relieving the inhibitory effect of Bcy1 (not shown). PKA phosphorylates both Msn2 and Msn4. The effect on Msn2 is to decrease its nuclear import and increase its nuclear export, rendering it less likely to activate its target genes, one of which is Msn4. Msn2 and Msn4 both repress cell growth and activate the stress response, but are not completely redundant and are therefore shown as regulating partially overlapping sets of stress-response target genes. See text for more details.

*TSL1* induction by stress is directly controlled by the paralogous transcription factors Msn2 and Msn4, which regulate it and other stress-response genes through stress-response (STRE) elements in their *cis*-regulatory regions [34–36]. The activities of Msn2 and Msn4 are controlled by intracellular cyclic AMP (cAMP) levels via the well-studied Ras/cAMP/protein kinase A (PKA) pathway [37, 38] (Fig 1). Intracellular cAMP levels are set by the balance between the adenylyl cyclase Cyr1 and the phosphodiesterases Pde1, which has low cAMP affinity, and Pde2, which has high cAMP affinity [39, 40]. The Ras proteins Ras1 and Ras2 are GTPases that can exist in GTP-bound and GDP-bound forms. In benign conditions with an abundance of the preferred carbon source, glucose, the GTP-bound form predominates. Under glucose starvation, the GTPase-activating proteins (GAPs) Ira1 and Ira2 stimulate the GTPase activity of Ras, causing a shift to the GDP-bound form. In its GTP-bound form, but not in its GDP-bound form, Ras stimulates Cyr1, causing cAMP levels to increase [41].

cAMP binds to Bcy1, the regulatory subunit of PKA, relieving an inhibitory association of Bcy1 with the catalytic subunit of PKA, encoded by one of three genes, *TPK1*, *TPK2* and *TPK3*. Deleting all three *TPK* genes is lethal, whereas deleting any one alone or any pair is not, implying redundancy [41]. However, substrate specificities of Tpk1, Tpk2 and Tpk3 do not completely overlap, and traits are known (including trehalose metabolism) for which the effects of deleting one *TPK* gene differ from the effects of deleting another, so redundancy is not absolute [42–44]. PKA phosphorylates Msn2 and Msn4, as well as other targets. PKA phosphorylates Msn2 at both its nuclear localization signal (NLS) and its nuclear export signal (NES), decreasing nuclear import and increasing nuclear export and thereby biasing Msn2 localization toward the cytoplasm [38, 45, 46]. PKA activity is opposed by protein phosphatase I, so when PKA activity is low, the NLS and NES of Msn2 are unphosphorylated and Msn2 localization is biased toward the nucleus, where it activates transcription of target genes [46–48]. Under severe glucose limitation, Msn2 spends more time in the nucleus but does not simply stay there. Instead, the Msn2 molecules within a cell show concerted movements in and out of the nucleus on a timescale of minutes, suggesting that PKA activity itself fluctuates even under constant conditions [46]. Msn4 is thought to behave similarly to Msn2, but the relationship between phosphorylation status and subcellular localization of Msn4 is not identical to that of Msn2 [38, 45]. *MSN2* and *MSN4* are partially redundant. *MSN2* is constitutively expressed, whereas *MSN4* is induced by stress (in an Msn2-dependent manner) [36], and the regulation of some target genes is dominated by one or the other [49].

Whereas the above-described pathway by which glucose starvation activates Msn2 and Msn4 is well known, the mechanism by which heat stress activates Msn2 and Msn4 is unknown [41, 50]. As glucose starvation does, heat stress leads to greater nuclear residency of Msn2 and Msn4, but this effect of heat stress is accompanied by Msn2 and Msn4 hyperphosphorylation, which can be blocked by addition of cAMP [51]. This result implies that sensing of heat stress does not go through PKA but instead involves other kinases [51]. Nonetheless, because Msn2 and Msn4 targets are implicated in protecting cells against a variety of environmental stresses, including nutrient starvation and heat shock [36], it could be that PKA is relevant to variability in survival of cells shifted from benign conditions to acute heat stress. That is, even though PKA does not transmit the signal of heat stress, cell-to-cell heterogeneity in PKA activity before acute heat stress could determine survival of such stress.

The possibility that fluctuations in PKA activity induce heterogeneity in Msn2 activity among unstressed cells that in turn creates heterogeneity in susceptibility to acute stress has been raised based on single-cell RNA sequencing of unstressed cells, which showed that a subset of unstressed cells had mildly activated Msn2-target genes; consistent with this observation, monitoring of cells under benign conditions for 80 min revealed a subset of cells in which Msn2 showed a peak of nuclear localization [52]. Further support for this possibility comes from a study that measured correlations between proteins in their variability across cells [53]. Specifically, genes encoding proteins that co-vary in expression across cells with the protein product of *PGM2*, a known target of Msn2 and Msn4, are highly enriched for STRE elements in their *cis*-regulatory regions; *TSL1* is one of these genes as are other stress-response genes [53]. Changing the gene dosage of Ras/cAMP/PKA pathway members, including Pde2, Ras2 and Ira2, alters the covariances between targets, suggesting that the Ras/cAMP/PKA pathway coordinates the heterogeneous expression of these targets [53].

Our hypothesis, therefore, is that under benign, glucose-replete conditions, cell-to-cell variability exists at some level in the Ras/cAMP/PKA pathway, and that this variability translates into variability in Msn2 and Msn4 activity and therefore variability in stress tolerance and growth rate. The variability in stress tolerance would come from variable expression of stress-response genes downstream of Msn2 or Msn4, such as *TSL1*. The mechanistic source of variability in growth rate is less clear, but might derive from the roles of Msn2 and Msn4 in entry into or exit from quiescence [41, 54, 55].

Here, we present tests of our hypothesis using genetic analysis of the Ras/cAMP/PKA pathway in combination with single-cell assays of cell growth, gene expression and stress tolerance. When testing our hypothesis, it was important to consider two possible alternative sources of variability in growth and stress tolerance: 1) so-called petite cells, which are deficient in respiration, and 2) DNA damage. Petite cells grow slowly, but do not represent nongenetic heterogeneity in that they result from mutations in the mitochondrial genome, complete loss of the mitochondrial genome, or mutations in nuclear genes encoding mitochondrial proteins [56–58]. Because petite cells also exhibit increased tolerance of some stresses, including heat shock [59–61], they could easily be confused with the slower-growing, stress-tolerant cells we aim to study [3]. The proportion of petites within a population varies considerably depending on strain genotype, ranging from 0.5% to 76% [62]. Even the same strain cultivated under identical conditions could have a large variance in petite frequency across experimental replicates [62], so petites add noise to measurements of growth-rate heterogeneity. For these reasons, we identified petite cells and excluded them from our analysis by integrating into the time-lapse microscopy assay the use of MitoTracker Red CMXRos, a non-toxic dye that differentially stains cells based on their mitochondrial inner membrane potentials [63]. We also created a rho^0^ petite strain in the genetic background used for our experiments, to serve as a control. rho^0^ petites have complete loss of the mitochondrial genome, are a common type of petite produced during normal cell division, and are indeed the final state of most petite lineages that start out with mitochondrial-genome mutations [64, 65].

DNA damage is the second possible alternative source of variability because it can both arrest growth and induce a stress response. Spontaneous DNA damage, such as base modification, base loss and strand breaks, happens during normal cell division [66–68]. Upon sensing DNA damage or replication-fork stalling, cells activate specific repair pathways to fix the corresponding problem [66, 68, 69]. In addition, a general DNA damage response (DDR) is activated to pause the current cell cycle [69–72], which would manifest as a slow-growing phenotype over the period of the DNA-damage repair process.

Two lines of evidence have suggested that DNA damage contributes to differences in growth and stress tolerance. First, *S*. *cerevisiae* cells with fewer divisions within a 4-h window have increased stress-response gene expression levels and a higher rate of formation of Rad52 foci, which indicate active double-strand break repair [73]. Cells within the top quartile of Rad52-GFP signal intensities show, on average, delayed division over the subsequent 45 min compared with the rest of the population [73]. However, this difference disappears after 2 h [73], and therefore might not be relevant to the heterogeneity in microcolony growth rates we observe, which persists through 10 h or more [3]. Second, spontaneous DNA damage can trigger entry into a so-called persister state, characterized by growth arrest and increased tolerance against various stresses [74]. However, persisters make up a very small fraction of the population (approximately 0.1%) and less than a quarter of them show sustained growth arrest for more than 5 h [74], so they do not appear to explain the nongenetic heterogeneity we observe, which involves a much larger subpopulation of cells that sustain slow growth over longer time periods. Therefore, although DNA damage impacts growth and stress tolerance, it is unlikely to explain the heterogeneity of growth rate and stress tolerance we observe. Nonetheless, we directly tested whether the DDR explains growth and stress-tolerance heterogeneity in our conditions. To do this, we used a protein fusion of GFP and Rnr3, an isoform of the large subunit of ribonucleotide reductase that is strongly induced as part of the DDR, independently of cell-cycle stage [75–77].

Further motivating our controls for petites and the DDR are known connections between petites and both DNA damage and the Ras/cAMP/PKA pathway. The existence of petites was neither monitored nor controlled for in the prior yeast studies examining DNA damage, growth and stress tolerance [73, 74]. However, petites could have been a major factor; for example, petites that have mutations in the gene encoding the mitochondrial protein frataxin induce a strong DDR because of increased genome instability [78]. The Ras/cAMP/PKA pathway has been reported to regulate mitochondrial biogenesis, enzyme content and chaperone expression [79–82]. *pde2* and *ira2* mutations have been reported to be petite-negative, meaning that petites cannot survive in the absence of these genes [83]. However, the exact mechanism explaining why petites cannot survive without Pde2 or Ira2 is not clear.

In this study, we separate nongenetic heterogeneity of growth rate and stress tolerance from petite formation and the DDR, and establish that the Ras/cAMP/PKA pathway, acting through Msn2 and Msn4, indeed regulates this nongenetic heterogeneity. This work not only contributes to our basic understanding of nongenetic heterogeneity but also points to an approach to defeating a bet-hedging strategy: increasing cAMP levels by administering a cAMP analog reduces the slower-growing fraction of the population and renders the population more susceptible to acute stress. This type of one-two punch might provide a useful treatment approach in cases where slow-growing subpopulations of pathogens or tumor cells cause relapse.

## Results

### Visualizing mitochondrial inner-membrane potential of founder cells identifies petite microcolonies without disturbing growth of non-petite microcolonies

**Fig 2:**
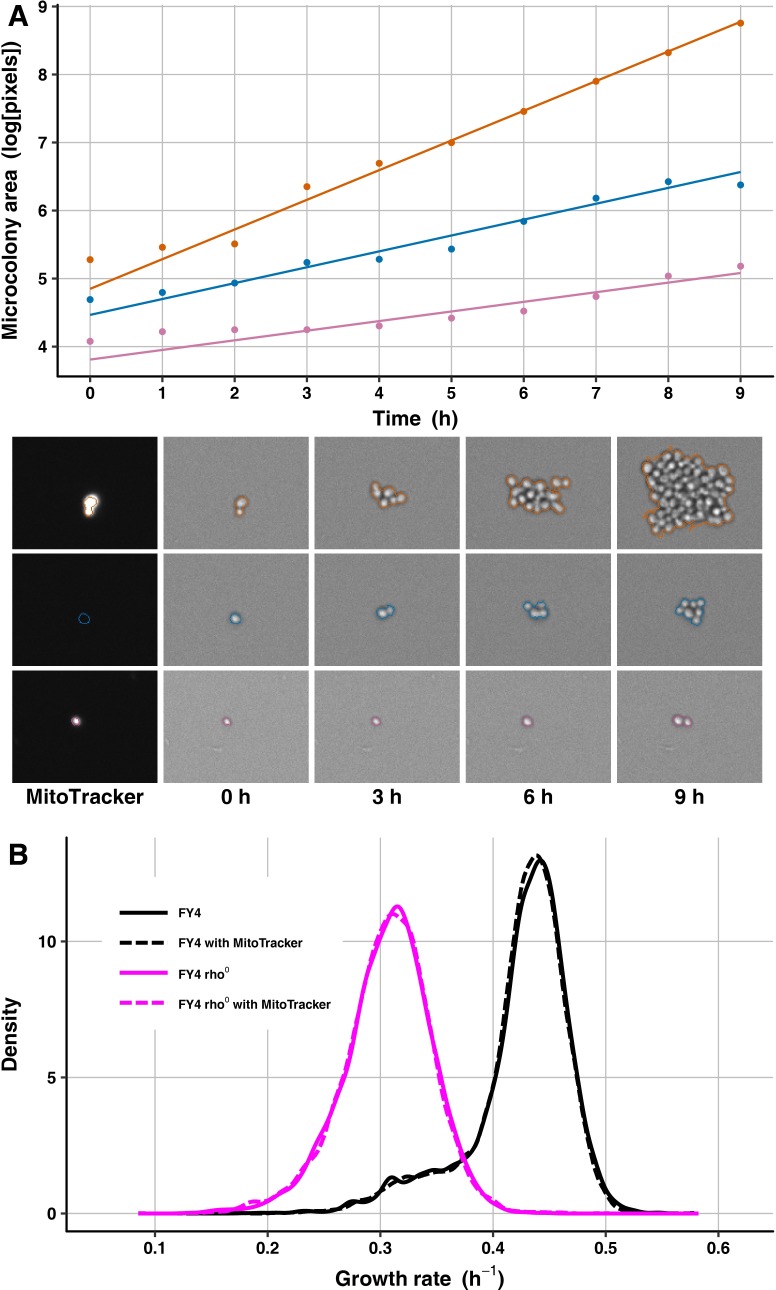
MitoTracker staining does not affect growth-rate distributions of wild-type or petite cell populations. (A) Microcolony areas were tracked through time to estimate growth rates, and MitoTracker staining was recorded at the first time point to identify petite cells. The changes in microcolony area over time are plotted for three FY4 microcolonies: a fast-growing colony (orange), a petite colony (blue), and a slow-growing non-petite colony (light purple). Solid lines are best-fit growth trajectories estimated as previously [6, 134]. Images below the plot show the corresponding microcolonies’ MitoTracker staining (left) and areas at several time points, with computed microcolony outlines indicated in the corresponding colors. (B) Shown is a density plot of microcolony growth rates for FY4 (black) and an FY4-derived rho^0^ petite strain (magenta) with (dashed line) and without (solid line) MitoTracker staining. Microcolony sample sizes: 6787 for FY4 without MitoTracker, 6966 for FY4 with MitoTracker, 6998 for petite without MitoTracker, 6883 for petite with MitoTracker.

We measured cell-growth heterogeneity within isogenic yeast populations using a previously established high-throughput microcolony growth-rate assay [3, 6, 33, 84] (Fig 2A). In brief, exponentially growing cell cultures are harvested at a density of ~10^6^ cells/ml (early log phase), diluted into fresh liquid medium so that ~4000 individual founder cells are dispensed per well of a concanavalin A-coated 96-well glass-bottom plate, then followed through time-lapse microscopy to capture changes in microcolony area over 10 h (see Methods). Concanavalin A is a lectin to which yeast cells adhere, so it causes cells to form discrete, monolayer microcolonies on the glass through the course of the experiment, despite being in liquid medium [3].

The typical microcolony growth-rate distribution for a wild-type, haploid strain of *S*. *cerevisiae* has a prominent peak of fast growth and a heavy left tail of slower-growing microcolonies, as shown in Fig 2B for the prototrophic haploid strain FY4 [85]. We used a prototrophic strain background, and when possible, clean genetic manipulations that leave no selectable markers in the genome (see Methods), because this assay is sensitive enough to detect growth differences between otherwise isogenic strains that differ only in which auxotrophic mutations they contain, even in rich medium that ostensibly renders the auxotrophies irrelevant [3].

The slower-growing tail of the microcolony growth-rate distribution might contain petites. To investigate this possibility and develop a means of excluding petites from our analyses, we cultured cells with MitoTracker Red CMXRos (hereafter called MitoTracker), a red-fluorescent dye that accumulates in actively respiring mitochondria, and analyzed red fluorescence at the first time point of microcolony growth (see Methods). To test the efficacy of this dye, we performed the microcolony growth-rate assay on FY4 cells, as well as on a rho^0^ petite strain derived from FY4 (see Methods), with and without MitoTracker. Under our conditions, MitoTracker does not alter growth of yeast cells, as the FY4 strain showed virtually indistinguishable growth-rate distributions with and without MitoTracker, as did the rho^0^ petite strain (Fig 2B). As expected, the rho^0^ strain had a lower mean growth rate than FY4, and its growth-rate distribution overlaps substantially with the left tail of the FY4 distribution (Fig 2B), suggesting that exclusion of petites is important for our analysis.

**Fig 3:**
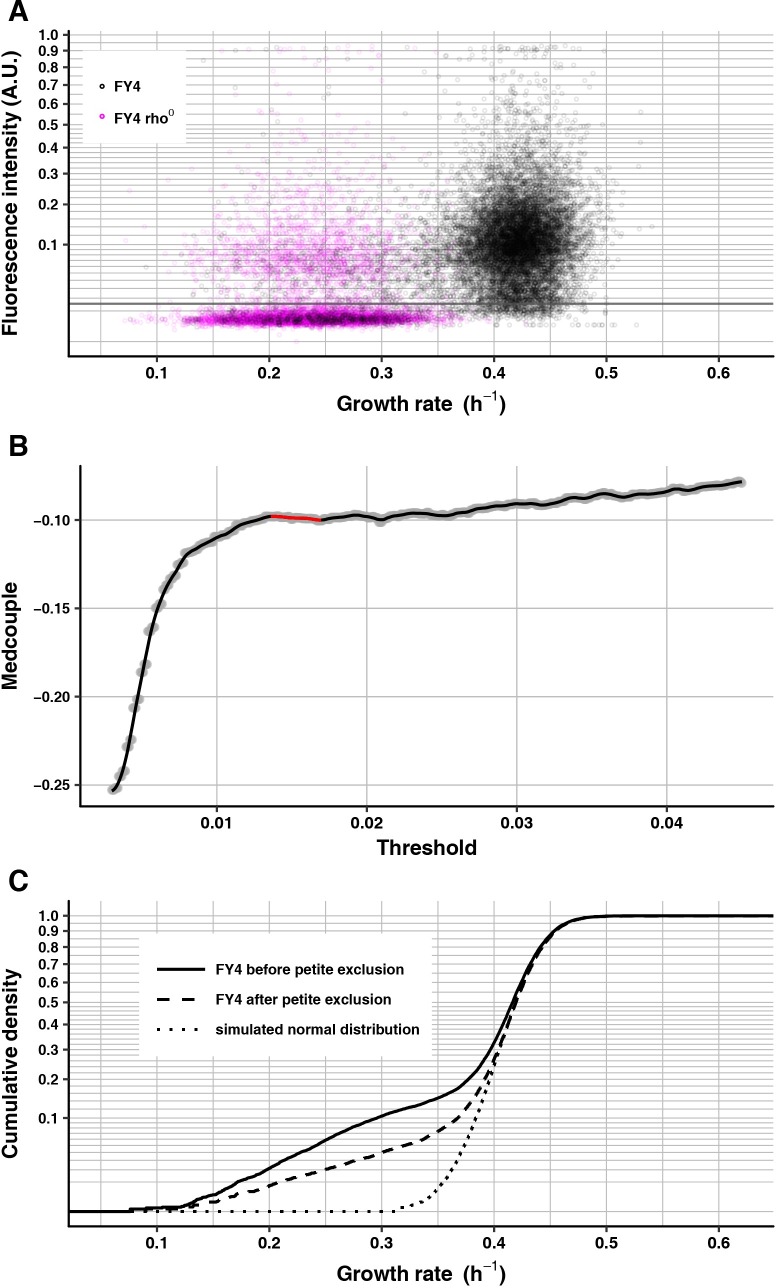
MitoTracker staining distinguishes FY4 cells from rho^0^ petites. (A) Scatter plot of mean MitoTracker fluorescence intensity (vertical axis) of FY4 (black, 13451 microcolonies) and FY4-derived rho^0^ petite (magenta, 7710 microcolonies) against microcolony growth rate (horizontal axis). The grey line shows the calculated threshold for FY4. MitoTracker fluorescence intensity is plotted on a square-root scale for a better view at the low-intensity end. (B) Medcouple of FY4 population after MitoTracker-based thresholding (vertical axis) plotted against corresponding MitoTracker fluorescence-intensity threshold. Grey dots are the calculated medcouples. The solid black line is the loess regression of medcouple on threshold. The highlighted red part shows the plateau region, determined in this case to span from 0.01360 to 0.01695, yielding a final threshold of 0.015275. (C) Growth-rate cumulative density curves of FY4 before (solid line) and after (dashed line) petite exclusion by MitoTracker thresholding. To provide a symmetrically tailed distribution for visual comparison, the dotted line shows simulated data from a normal distribution. This normal distribution is centered at the median of the “after” distribution, and has standard deviation equal to the magnitude of the difference between this median and the quantile of the “after” distribution corresponding to the standard normal cumulative distribution function evaluated at 1 (~84%). The vertical axis is on a square-root scale for a better view of the slower-growing tails of each distribution.

To exclude petites, we used fluorescence intensities of MitoTracker staining of rho^0^ petite microcolonies to determine a threshold under which to remove microcolonies from analyses of other strains on the same experimental plate. Most rho^0^ petite microcolonies have very low MitoTracker intensities relative to most FY4 microcolonies (Fig 2A), but a small proportion of rho^0^ petite microcolonies have high MitoTracker intensities, perhaps because they are mutants with increased mitochondrial inner-membrane potential. For FY4, a small proportion of microcolonies have low MitoTracker intensities and reduced growth rates, which overlap with the majority of the rho^0^ petite population. The remaining FY4 microcolonies have relatively high MitoTracker intensities, but there is a small positive correlation with growth rate; although slower-growing cells are therefore more likely to have comparatively low MitoTracker staining, this staining is still higher than that of most of the petite cells (Fig 3A).

Because the MitoTracker staining levels of FY4 cells and the rho^0^ petites overlap and because there is a slight positive correlation between MitoTracker staining and growth rate, the decision of where to set a stain-intensity threshold for excluding petites is critical. If the threshold is too low, it will not sufficiently filter out petite cells, whereas if it is too high, it could filter out slow growers in a biased way. To assess how different thresholds alter the apparent non-petite growth-rate distribution, we used the medcouple, a measure of the skewness of a distribution that is robust against outliers and is location- and scale-invariant [86]. For subsequent comparisons of non-petite growth-rate distributions between different genotypes or conditions, an added advantage of the medcouple is that its values can be compared statistically with a Z-test [86].

We calculated the medcouple across a wide range of thresholds. The medcouple should be negative when there is a skew to the left (i.e., a heavier tail of slower-growing cells than of faster-growing cells). We reasoned that, when a threshold is low and located in the range of petite intensities, increasing the threshold would increase the medcouple by preferentially removing petite cells and thereby reducing the left tail. Likewise, when a threshold is high and located in the range of normal-cell intensities, increasing the threshold would also increase the medcouple by disproportionately removing slower-growing cells because of the positive correlation between MitoTracker intensity and growth rate, which is especially evident in the growth-rate range between 0.3 and 0.4 h^–1^ (Fig 3A). Between these two phases of increasing medcouples should lie a plateau of medcouples corresponding to thresholds that filter out most petites yet do not disproportionately remove many slower-growing non-petites.

In plots of medcouples against corresponding MitoTracker-intensity thresholds that span a wide range, the three phases appear as expected (Fig 3B). Removing petite microcolonies by setting the threshold in the center of the middle plateau phase, as expected, reduces the left tail of the growth-rate distribution but, also as expected, the distribution does remain asymmetric, with a substantial fraction of slower-growing cells (Fig 3C). Unless otherwise specified, all microcolony growth-rate analyses below used this MitoTracker approach to exclude petites. When multiple strains or conditions were assayed in the same experimental replicate, appropriate thresholds were calculated for each strain separately, then a single threshold was set for the entire replicate by taking the mean value of these thresholds.

To confirm the robustness of any conclusions we drew, we repeated all analyses using thresholds set at 90% or 110% of the originally selected common threshold. We also repeated all analyses using the separate strain- or condition-specific thresholds rather than a common threshold, in case threshold differences reflect actual biological differences between strains or conditions in MitoTracker staining rather than experimental noise. Results were consistent across analyses with these different thresholds, so for simplicity of presentation we report below the results of using the threshold corresponding to the mean of the plateau centers of each strain or condition.

### DNA damage does not explain the existence of the slower-growing cell population

To test the possible relationship between DNA damage and slow growth, we used a strain carrying two reporter genes encoding fluorescent-protein fusions: *HSP12-mCherry* and *RNR3-GFP*. *HSP12* is induced by environmental stresses and by DNA damage, and has been proposed to be a marker of yeast persister cells [74]. Hsp12 expression highly correlates with that of Tsl1, and both *HSP12* and *TSL1* are targets of Msn2 [53, 74]. *RNR3* is induced as part of the DDR and is not a target of Msn2 [35, 74]. We first tested whether Hsp12 behaves similarly to Tsl1 in growing microcolonies and indeed it does. We cultured the dual-reporter strain to early-log phase and measured mCherry intensity and growth in microcolonies. As is seen with a Tsl1 reporter [3] (and this study; see below), Hsp12-mCherry expression correlates negatively with microcolony growth rate (S1A Fig).

The negative relationship between growth and Hsp12 (or Tsl1) expression might be caused by DNA damage or by something else, such as DNA damage-independent regulation of growth and expression by Msn2. We therefore next tested more specifically how the DDR correlates with differences in growth rate. We performed this test by collecting fractions of cells with different expression levels of the DDR reporter Rnr3-GFP and measuring the distributions of their microcolony growth rates. We did not measure Rnr3-GFP fluorescence on the microscope the same way as we did for Tsl1 and Hsp12, because Rnr3 signal is not detectable in cells from early log-phase culture. Instead, we used fluorescence-activated cell sorting (FACS) to sort the early log-phase population according to the fluorescence intensity of Rnr3-GFP. A quantile-quantile plot (S1B Fig) comparing the fluorescence-intensity distribution of Rnr3-GFP to that of FY4 (serving as a no-GFP control) shows two deviations from identity: 1) the plot is parallel to the null-expectation diagonal except for an upward shift for Rnr3-GFP, suggesting that Rnr3-GFP has a basal level of fluorescence possibly caused by leaky expression; and 2) the plot shows an extended tail of very intense Rnr3-GFP cells, which we infer to have activated the DDR. Linear regression of Rnr3-GFP quantiles on control FY4 quantiles, using data from the 10% to 25% quantiles, where the plot appears largely parallel to the diagonal, yields a slope near one (1.016), suggesting that indeed the Rnr3-GFP fluorescence distribution overlaps with that of the no-GFP control, when correcting for putative leaky expression and excluding the high-expressing tail.

We stringently extracted DDR-activated cells by gating at a fluorescence intensity that selects the most intense ~0.2% of the total population. This top 0.2% bin shows a significant increase in slower-growing cells (medcouple Z-test *P* = 1.75 X 10^−42^) but this increase is more than offset by a shift in the mode of the growth-rate distribution toward faster growth (S1C Fig), consistent with the previous finding that cells with high Rad52-GFP signal intensities show slower division times on average than those with low Rad52-GFP signal intensities when assayed in a short time window (45 min) but not after 2 h [73]. The increase in slower-growing cells suggests that DNA damage does cause some cells to arrest growth over a relatively long time-frame of several hours, which is consistent with the finding that some high-Hsp12, slow-growing “persisters” have long-lived Rad52 foci [74]. However, our results imply that DNA damage does not make a meaningful contribution to the broad slower-growing tail of the typical wild-type growth-rate distribution, because cells whose growth is slowed in an enduring way by DNA damage make up such a small minority of the population (much less than 0.1%). Moreover, this minority subpopulation might be even smaller than it appears: it likely contains petites because respiration-deficient cells have higher genome instability [78]; we could not use MitoTracker to exclude petites in this experiment because Hsp12-mCherry signal would interfere.

To rule out the possibility that a less stringent FACS gating would yield different results, we repeated the Rnr3-GFP sorting experiment with GFP-intensity gates set to capture different bins of GFP intensity (with 0% being the most intense): 0–2%, 5–7%, 10–12% and 20–25%. We chose 20–25% as the lowest bin because cells within the 20–25% bin show no evidence of having activated the DDR: as indicated in S1B Fig, the 20–25% bin is located within the region where the quantile-quantile plot coincides with the linear-regression line. The 0–2% bin behaves similarly to the 0–0.2% bin, in that it shows an increase in the slower-growing tail as well as an increase in the mode of the growth-rate distribution, whereas the growth-rate distributions of the remaining bins are very similar in shape to that of the ungated population but have modes that exceed or equal that of the ungated population (S1D Fig). The results with different Rnr3-GFP intensity gates therefore support the original conclusion that DNA damage does not explain the existence of the broad slower-growing tail of the wild-type growth-rate distribution. Correspondingly, we conclude that the negative correlation between growth and Hsp12 (or Tsl1) expression is largely independent of DNA damage.

### Increasing intracellular cAMP levels reduces the abundance of slower-growing cells and reduces heterogeneous Tsl1 expression

**Fig 4:**
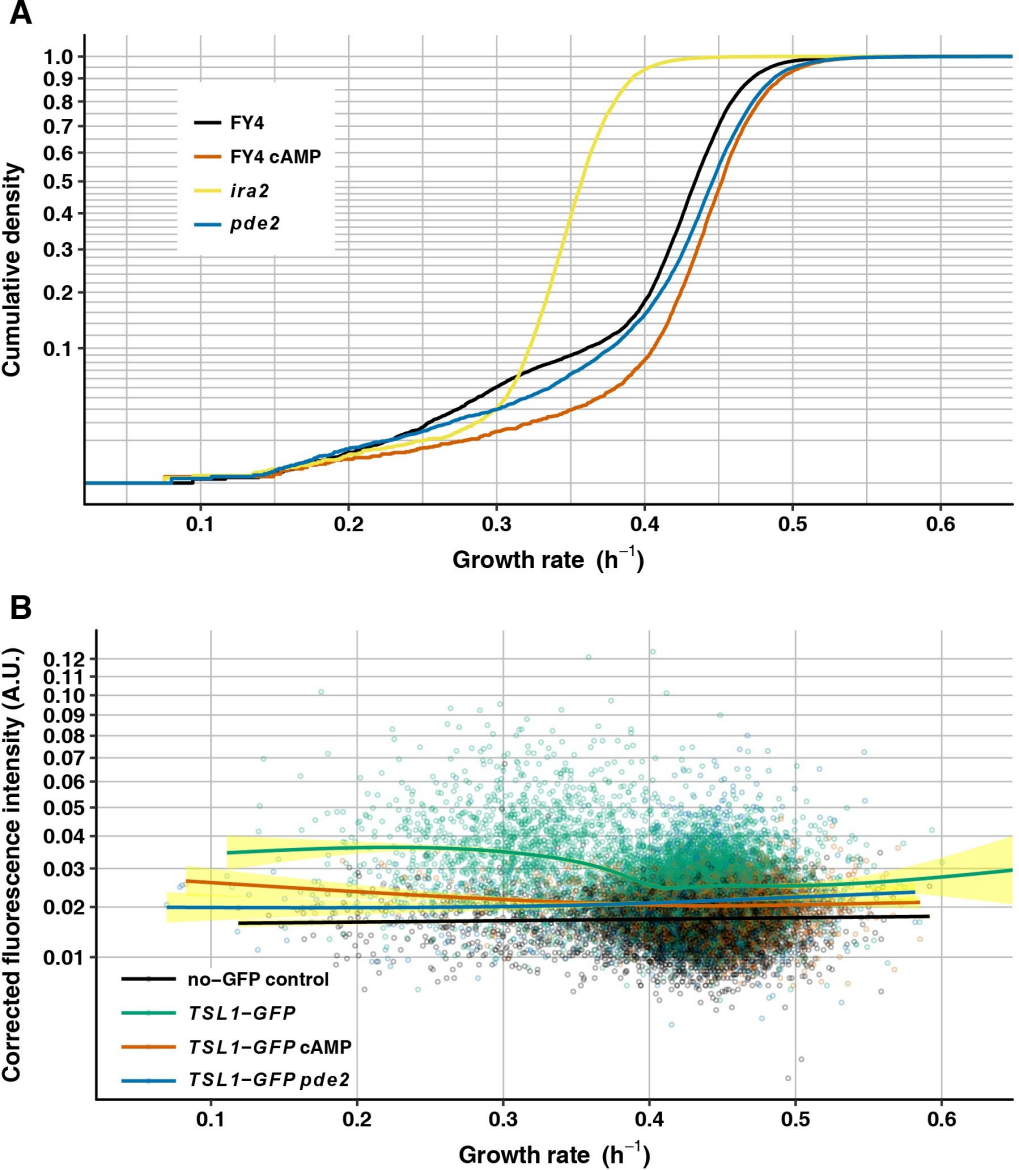
Intracellular cAMP controls nongenetic heterogeneity. (A) Growth-rate cumulative density curves of FY4 (black, 11900 microcolonies), FY4 cultivated with 15 mM 8-bromo-cAMP (orange, 4510 microcolonies), *ira2* (yellow, 6495 microcolonies) and *pde2* (blue, 8666 microcolonies). Vertical axis is on a square-root scale for a better view of the slower-growing tail of each distribution. (B) Mean GFP fluorescence intensity—corrected by subtracting local background fluorescence then by subtracting the minimum value for the entire experiment, to avoid negative values (see Methods, vertical axis)—is plotted against microcolony growth rate (horizontal axis) for FY4 no-GFP control (black, 7340 microcolonies), *TSL1-GFP* (green, 6912 microcolonies), *TSL1-GFP* cultivated with 15 mM 8-bromo-cAMP (orange, 3730 microcolonies) and *TSL1-GFP pde2* (blue, 1778 microcolonies). Each solid line is the fit to a generalized additive model with cubic spline smoother, with 95% confidence interval shown in yellow. Vertical axis is on a square-root scale for a better view at the low-intensity end.

To test the role of the Ras/cAMP/PKA pathway in nongenetic heterogeneity of growth rate, we first performed genetic and chemical manipulations that increase intracellular cAMP levels. Intracellular cAMP levels can be increased by *ira2* or *pde2* loss-of-function mutations [87, 88], or by directly providing cAMP in the culture medium in the form of 8-bromo-cAMP, a cell-permeable cAMP analog that has higher resistance to phosphodiesterase activity [89]. We compared the growth-rate distribution of the wild-type FY4 strain with that of FY4 cultured in the presence of 15 mM 8-bromo-cAMP and with those of FY4-derived strains with *pde2* or *ira2* deletions (Fig 4A). 8-bromo-cAMP significantly increases the population mean growth rate (0.424 h^–1^ without 8-bromo-cAMP vs. 0.448 h^–1^ with 8-bromo-cAMP, Wilcoxon-Mann-Whitney test, *P* < 2.2 X 10^−16^), and reduces the abundance of slower-growing cells (less left skew, medcouple Z-test *P* = 4.52 X 10^−4^). As 8-bromo-cAMP does, the *ira2* mutation renders the growth-rate distribution less skewed (medcouple Z-test *P* = 1.27 X 10^−10^). However, relative to FY4 cells *ira2* cells grow much slower on average (0.356 h^–1^, Wilcoxon-Mann-Whitney test, *P* < 2.2 X 10^−16^). This decrease in mean growth rate might be caused by the cAMP-independent requirement for Ira2 in glycerophosphoinositol production and transport [90]. Relative to FY4 the mean growth rate of the *pde2*-mutant strain is higher (0.437 h^–1^, Wilcoxon-Mann-Whitney test, *P* < 2.2 X 10^−16^). The shape of the left tail of the *pde2*-mutant growth-rate distribution is also altered relative to FY4. As shown in Fig 4A, the *pde2* mutant has a reduced abundance of the slowest-growing cells but also an increased abundance of cells with intermediate growth rate (i.e., the distribution has a heavy shoulder in that its cumulative density curve has a more gentle slope than that of FY4 in the range of growth rates between 0.4 and 0.5). This altered shape results in a nonsignificant difference in medcouple between FY4 and *pde2* mutant (medcouple Z-test *P* = 0.492). It is unclear why the shape of the growth-rate distribution differs between the addition of 8-bromo-cAMP and the *pde2* deletion, although we note that the effects of *pde2* deletion appear to be sensitive to the exact nature of the deletion, suggesting that mutations might have differential effects on local chromatin structure and neighboring gene activity. Specifically, it was previously reported that *pde2*-deletion mutants cannot survive as petites [83]; when we deleted *pde2* in a similar way to this previous study by replacing the gene with a selectable marker we found the same result, but when we subsequently removed the marker sequences to leave a clean deletion we found that petites can form. Thus, our attempt at the most precise control (clean deletion without marker sequences) might have produced an artifactual result.

To test the role of the Ras/cAMP/PKA pathway in nongenetic heterogeneity of Tsl1 expression we measured fluorescence intensity of a Tsl1-GFP fusion protein during microcolony growth of FY4, FY4 cultured with 15 mM 8-bromo-cAMP, and the FY4-derived *pde2*-deletion mutant. The wild-type strain encoding the Tsl1-GFP fusion protein showed the expected negative correlation between microcolony growth rate and Tsl1-GFP fluorescence intensity [3] (Fig 4B, S2 Fig). In striking contrast, for both FY4 cultured with 8-bromo-cAMP and the *pde2* mutant, across growth rates Tsl1-GFP fluorescence was at close to baseline levels. That is, these manipulations that increase intracellular cAMP levels largely abolished heterogeneous Tsl1 expression and they did so by reducing expression in the slower-growing cells (rather than by increasing expression in the faster-growing cells).

The data presented in Fig 3A and Fig 4B (and S2B Fig) provide additional confirmation that our MitoTracker-based identification of petites is indeed removing the confounding effect of petites. As noted above, elimination of petites by MitoTracker staining, although effective, is not perfect. In Fig 3A, the proportion of microcolonies in the wild-type FY4 population that fall below the MitoTracker threshold is 12.45% and the proportion of microcolonies in the FY4 rho^0^ petite population that exceed the MitoTracker threshold (which was set based solely on the wild-type FY4 population’s data) is 21.79%. We can estimate the extent to which petites contaminate the after-thresholding wild-type FY4 data set by assuming that the proportion of such contaminating petites (relative to the total number of petites) matches the proportion of FY4 rho^0^ petites that exceed the MitoTracker threshold. Thus, the estimated total petite frequency is 15.92% and the estimated amount of contamination of the after-threshold data by petites is 3.96%. This estimate of contamination is likely an over-estimate because: 1) the competitive disadvantage of rho^0^ petites is known to be suppressible by mutations that increase inner-membrane potential [91], and rho^0^ petites have genome instability and therefore increased mutation rate [65]; and 2) the MitoTracker threshold was set without using the FY4 rho^0^ data. On this latter point, we note that we have found that including rho^0^ data in the threshold calculation increases the calculated threshold, so that the proportion of rho^0^ microcolonies that exceed the threshold decreases. That is, although an increase in petite frequency is expected to increase the amount of contamination, this increase is mitigated by a resulting increase in the MitoTracker threshold. To simulate a “worst-case” scenario of high petite frequency in a particular experiment, we sampled FY4 rho^0^ microcolonies to add to the wild-type FY4 data set at a final proportion of 38%. We chose 38% because it is approximately the petite frequency mean plus twice the standard deviation, as estimated by the proportion of microcolonies below the MitoTracker threshold in six biological replicate experiments on the wild-type FY4 genotype. This value of 38% also exceeds the maximum petite frequency we observed for this genotype by direct scoring of petite colonies in 20 independent experiments (range for these 20 experiments: 1.59%– 27.9%). The resulting contamination estimate, based on 100 such random samplings, has mean 2.34% and standard deviation 0.56%.

We can compare this expected amount of contamination with the proportion of slower-growing, high-Tsl1 microcolonies, as estimated from the data presented in Fig 4B and S2B Fig. Examination of Fig 4B and S2B Fig suggests there are two clusters of microcolonies in the wild-type genetic background: one containing faster-growing, low-Tsl1 microcolonies and one containing slower-growing, high-Tsl1 microcolonies. We used partitioning around medoids to separate the data into two clusters and indeed the two expected clusters emerge (with one medoid at growth rate 0.436 h^–1^ and Tsl1-GFP fluorescence intensity 0.0249 and the other medoid at growth rate 0.317 h^–1^ and Tsl1-GFP fluorescence intensity 0.0366) (S3A Fig). The slower-growing, higher-Tsl1 cluster contains 20.4% of the microcolonies, so contamination by fewer than 4% petites would not substantially change the appearance of the plot. Moreover, we can flip the analysis around and select the microcolonies from this strain that passed the MitoTracker threshold but are most likely to be petites (by selecting those microcolonies in the lowest 3% of MitoTracker staining) and ask where these points fall on the plot. As expected, these microcolonies are biased toward being slower growing, but they are not biased toward higher Tsl1-GFP fluorescence (S3B Fig). Even for the microcolonies that did not pass the MitoTracker threshold and are therefore the most likely to be petites, there is no evidence of bias toward higher Tsl1-GFP fluorescence despite an increased bias toward being slower growing (S3B Fig). Therefore, an erroneous inclusion of petites in the wild-type Tsl1-GFP data could not explain the dramatic change in the relationship between Tsl1-GFP and growth rate upon addition of 8-bromo-cAMP or *pde2* mutation.

### Intracellular cAMP levels are lower in cells naturally expressing high levels of Tsl1 under benign conditions

If intracellular cAMP levels control heterogeneity in growth and stress-response gene expression, then not only should manipulations of cAMP levels have predicted effects, but also cAMP levels should differ between unmanipulated cells. In particular, slower-growing cells should have lower cAMP levels. To test this prediction, we used FACS on the FY4-derived *TSL1-GFP* strain to obtain subpopulations of cells expressing high (top 10%) or low (bottom 50%) levels of Tsl1, and then measured cAMP concentration in cell extracts (see Methods). These measurements support the prediction. The cAMP concentration of the high-Tsl1 fraction is approximately 27% lower than that of the low-Tsl1 fraction, with the difference estimated as 0.832±0.217 pmol/10^7^ cells (*P* = 0.0007 by likelihood ratio test of nested mixed-effect linear models that take into account technical and biological replicates and do or do not include a fixed effect for fraction membership).

Because the experiments to measure cAMP were performed without MitoTracker-based exclusion of petites, we additionally confirmed that the results were not somehow biased by petite cells. We measured petite frequencies by plating on standard YPD agar plates approximately 1000 cells from each member of five replicate pairs of high-Tsl1 and low-Tsl1 FACS samples, and staining with 2,3,5-triphenyltetrazolium chloride (TTC) to reveal colonies with functional mitochondria, which appear red rather than white (see Methods). There is no significant difference in petite frequency between the high-Tsl1 and low-Tsl1 fractions (15.3% vs. 14.4%, respectively; paired t-test on arcsine-transformed frequencies *P* = 0.685).

### Msn2 nuclear occupancy is heterogeneous under benign conditions and correlates with growth rate at the single-cell level

The difference in intracellular cAMP concentration between the Tsl1-sorted subpopulations enriched for slower- or faster- growing cells suggests that heterogeneity in cAMP levels exists and might correlate with growth at the single-cell level. It would be ideal to quantify intracellular cAMP at the single-cell level in the context of the microcolony growth assay. A fluorescence resonance energy transfer (FRET) cAMP sensor successfully reports the cAMP increase in starved cells upon glucose addition [92]. We tested this FRET sensor in our strain background, and unfortunately it did not have sufficient signal-to-noise ratio to measure cAMP heterogeneity in cells under benign conditions, which is not entirely surprising given that this heterogeneity is likely of considerably lower magnitude than the concentration change after adding glucose to starved cells. Moreover, even if a less-noisy sensor could be found, we would worry that a kind of observer effect would pertain: by measuring cAMP the sensor would take cAMP out of the natural system and thereby perturb the system’s function. Our solution is instead to use what has been demonstrated to be an endogenous reporter of cAMP concentration, by virtue of the effect of cAMP on PKA activity: nuclear localization of Msn2 [38]. Higher levels of cAMP are associated with higher PKA activity and therefore lower nuclear occupancy of Msn2, whereas lower levels of cAMP are associated with lower PKA activity and therefore higher nuclear occupancy of Msn2.

**Fig 5:**
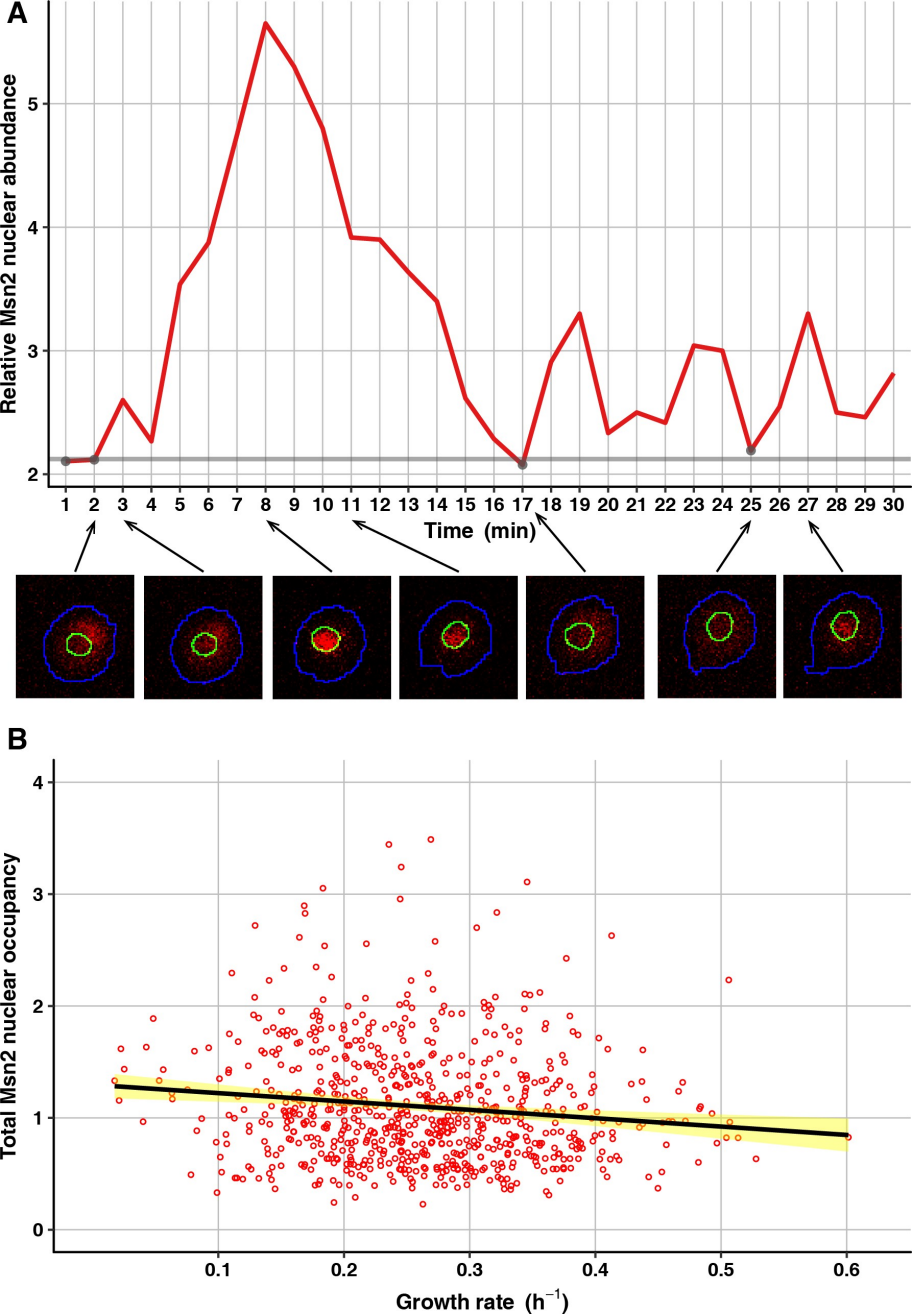
Msn2 subcellular localization dynamics negatively correlate with microcolony growth rate. (A) An example tracking of Msn2 localization dynamics in a single cell. Plotted is relative Msn2 nuclear abundance across 30 time points at 1-min intervals. Corresponding cell images for seven of the time points are shown below, with Msn2-mRuby2 signal in red and the determined cell and nuclear boundaries in blue and green, respectively. The time points with the four lowest values (gray points) were used to determine the baseline (horizontal gray line) to be subtracted from each relative abundance for the other time points. These corrected abundances were then summed to generate the total Msn2 nuclear occupancy. (B) Total Msn2 nuclear occupancy for each cell (total sample size 720 cells) is plotted against growth of the microcolony it founded. The solid black line shows the linear regression, with 95% confidence interval shown in yellow.

To test for an association between Msn2 nuclear occupancy and growth, we conducted experiments in which we monitored Msn2 localization at 1-min intervals in individual cells for 30 min, then tracked growth of the microcolonies produced by those cells as usual (see Methods). For these experiments, we constructed a strain in the FY4 genetic background expressing a fusion of Msn2 with the red-fluorescent protein mRuby2 (*MSN2-mRuby2*), as well as a fusion of the core histone Htb2 with GFP (*HTB2-GFP*, to mark nuclei) and a fusion of Tsl1 with the teal-fluorescent protein mTFP1 (*TSL1-mTFP1*, to sort cells by FACS). FACS sorting by Tsl1 expression was used because the imaging at 1-min intervals considerably reduces the throughput of the experiment, so it was necessary to enrich for slower-growing cells to get a sufficient representation of them. At each of the 30 time points for imaging Msn2 localization, we segmented each cell into nucleus and cytoplasm using the Htb2-GFP signal, then computed relative Msn2 nuclear abundance as the ratio between the median nuclear intensity of mRuby2 and the median cytoplasmic intensity of mRuby2 (Fig 5A, see Methods). From these relative abundances, we then computed for each cell a measure we call total nuclear occupancy. This measure is calculated by first identifying a baseline relative nuclear abundance as the mean of the four lowest relative abundances, then summing the differences between this baseline and the relative abundance at each other time point, then dividing by the number of such time points (because not all cells are faithfully tracked for all 30 time points). Setting the baseline in this way, for each cell, is important because of technical variation in fluorescence intensity within and between images. Changing the baseline-setting procedure to include the one, two, three or five lowest relative abundances, rather than the four lowest, has an extremely minor effect on the computed total nuclear occupancies and does not change any of the conclusions reported here.

The total nuclear occupancy measure is somewhat noisy, as it is based on a small number of time points (capturing a few nuclear entry events per cell at most) and because of the limitation of imaging cells in the context of the microcolony assay (i.e., not in a plane constrained by a microfluidic chamber or a slide and coverslip). Nonetheless, as predicted, a cell’s total nuclear occupancy of Msn2 negatively correlates with subsequent growth of the microcolony founded by that cell (Fig 5B). This result suggests that cell-to-cell differences in cAMP concentration do indeed underlie differences in growth. Of course, an unknown factor other than cAMP concentration might be determining Msn2 localization under these conditions, but given the cAMP concentration difference we saw in the bulk subpopulations of slower- and faster-growing cells and given the well-studied effect of cAMP concentration on PKA activity and Msn2 localization, we consider cAMP the most likely candidate at this point. And at the very least, our results demonstrate that Msn2 subcellular localization dynamics matter not only during responses to stress [46], but also under benign conditions.

### The PKA-responsive transcription factors Msn2 and Msn4 are required for nongenetic heterogeneity

Having shown that cAMP levels are relevant to nongenetic heterogeneity in growth and Tsl1 expression, and that heterogeneity of Msn2 activity (likely caused by heterogeneity of cAMP levels acting through PKA) correlates with growth, we next sought to test a causative relationship between Msn2 (and Msn4) and nongenetic heterogeneity. We therefore tested whether removing Msn2 or Msn4 reduces the abundances of slower-growing cells and high-Tsl1 cells.

**Fig 6:**
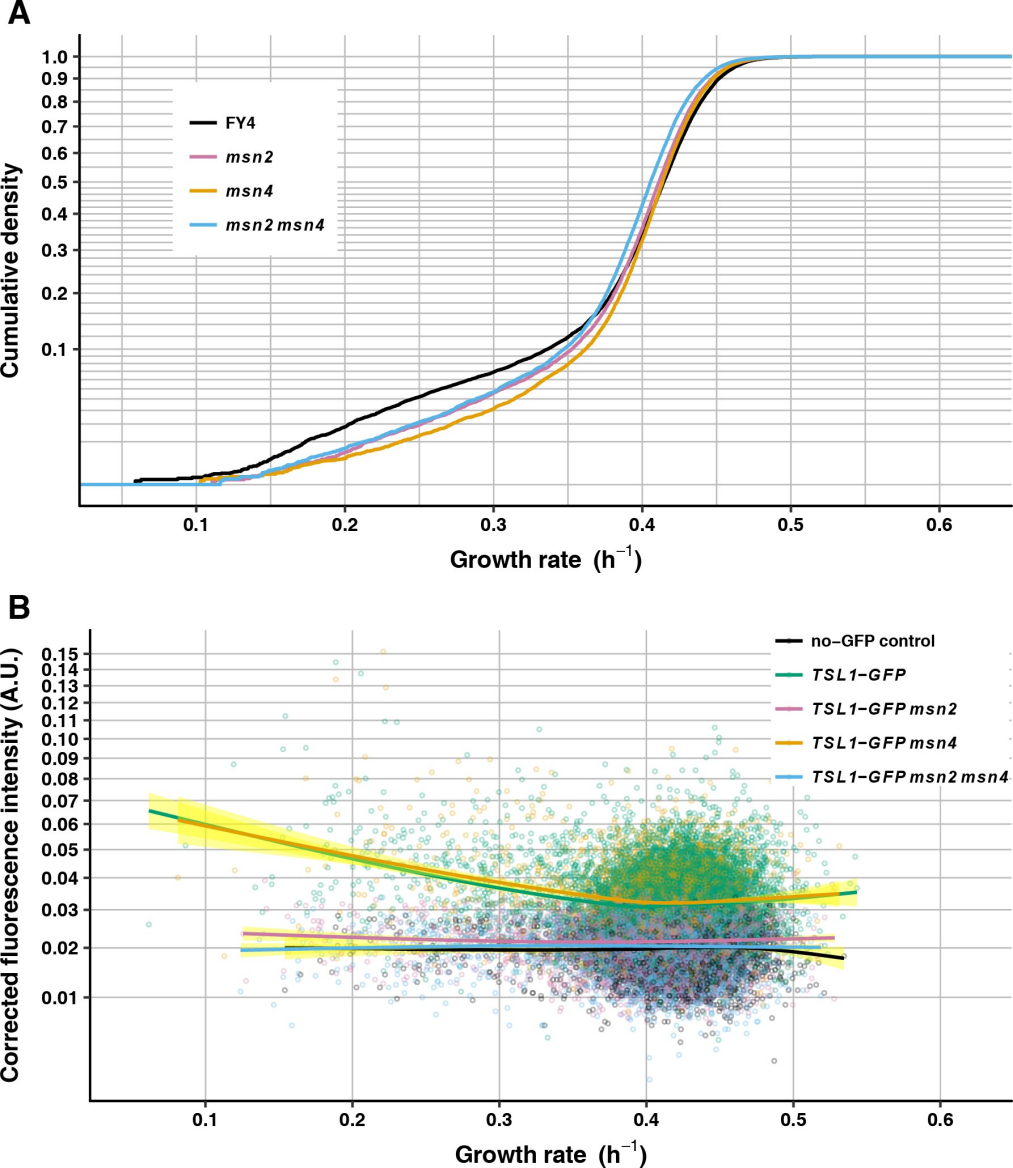
Msn2 and Msn4 are required for nongenetic heterogeneity. (A) Growth-rate cumulative density curves of FY4 (black, 14470 microcolonies), *msn2* (light purple, 14899 microcolonies), *msn4* (light orange, 11729 microcolonies) and *msn2 msn4* (light blue, 11894 microcolonies). Vertical axis is on a square-root scale for a better view of the slower-growing tail of each distribution. (B) Mean GFP fluorescence intensity—corrected by subtracting local background fluorescence then by subtracting the minimum value for the entire experiment, to avoid negative values (see Methods, vertical axis)—is plotted against microcolony growth rate for FY4 no-GFP control (black, 3915 microcolonies), *TSL1-GFP* (green, 10531 microcolonies), *TSL1-GFP msn2* (light purple, 6460 microcolonies), *TSL1-GFP msn4* (light orange, 3724 microcolonies), and *TSL1-GFP msn2 msn4* (light blue, 5621 microcolonies). Each solid line is the fit to a generalized additive model with cubic spline smoother, with 95% confidence interval shown in yellow. Vertical axis is on a square-root scale for a better view at the low-intensity end.

We compared the growth-rate distribution of the FY4 wild type with those of FY4-derived *msn2*-deletion mutants, *msn4*-deletion mutants, and the *msn2 msn4* double deletion. Relative to the growth-rate distribution of FY4, the growth-rate distribution of *msn2*-mutant cells has significantly fewer slower-growing cells (medcouple Z-test *P* = 5.32 X 10^−5^), as do the growth-rate distribution of *msn4*-mutant cells (medcouple Z-test *P* = 5.19 X 10^−7^) and the growth-rate distribution of *msn2 msn4* double-mutant cells (medcouple Z-test *P* = 1.84 X 10^−6^) (Fig 6A). There is no significant difference in skew between any pair of these mutant genotypes (medcouple Z-test, *msn2* vs. *msn4*: *P* = 0.105; *msn2* vs. *msn2 msn4*: *P* = 0.163; *msn4* vs. *msn2 msn4 P* = 0.397). These results imply that Msn2 and Msn4 are each necessary for nongenetic heterogeneity by virtue of increasing the proportion of slower-growing cells.

Msn2, but not Msn4, is also required for heterogeneous expression of Tsl1 under benign conditions. The *msn2*-deletion mutant and the *msn2 msn4* double-deletion mutant qualitatively gave the same result as did the *pde2*-deletion mutant and the FY4 cells cultured with 8-bromo-cAMP: across growth rates Tsl1-GFP fluorescence was at close to baseline levels (Fig 6B, S4 Fig). By contrast, the *msn4*-deletion mutant qualitatively gave the same result as did the FY4 wild type: a negative correlation between microcolony growth rate and Tsl1-GFP fluorescence intensity (Fig 6B, S4 Fig). These results establish Msn2 as a key regulator of heterogeneous gene expression under benign conditions, and provide additional evidence that slow growth and the stress response are not inextricably linked, in that *msn4* mutants maintain heterogeneous Tsl1 expression but show reduced abundance of slower-growing cells.

### PKA activity impacts nongenetic heterogeneity

The effects on the growth-rate distribution of manipulations of cAMP levels and of *msn2* and *msn4* mutations are consistent with each other and predict that raising PKA activity would cause there to be fewer slower-growing cells whereas lowering PKA activity would skew the growth-rate distribution toward slow growth. One way to test this prediction is to study mutations in the genes encoding PKA subunits. However, the effects of mutations in PKA components are highly pleiotropic and are typically difficult to interpret, because: 1) PKA signaling influences many cellular processes, including carbon usage, stress resistance and ribosome biogenesis; 2) PKA activity is embedded in nutrient- and stress-sensing networks with complex cross-talk and feedbacks; and 3) the three Tpk catalytic subunits are not completely redundant [36, 38, 41, 93–96]. Indeed, we find that PKA mutations have effects opposite to the simple prediction. The growth-rate distributions of *tpk1* or *tpk2* deletion mutants have reduced left tails relative to FY4, whereas the growth-rate distribution of a *bcy1* deletion mutant is heavily skewed toward slow growth (S5 Fig). Because this contrary result might be caused by complex feedbacks, we sought to test the role of PKA activity in nongenetic heterogeneity by assaying cells upon mild perturbation of this activity.

**Fig 7:**
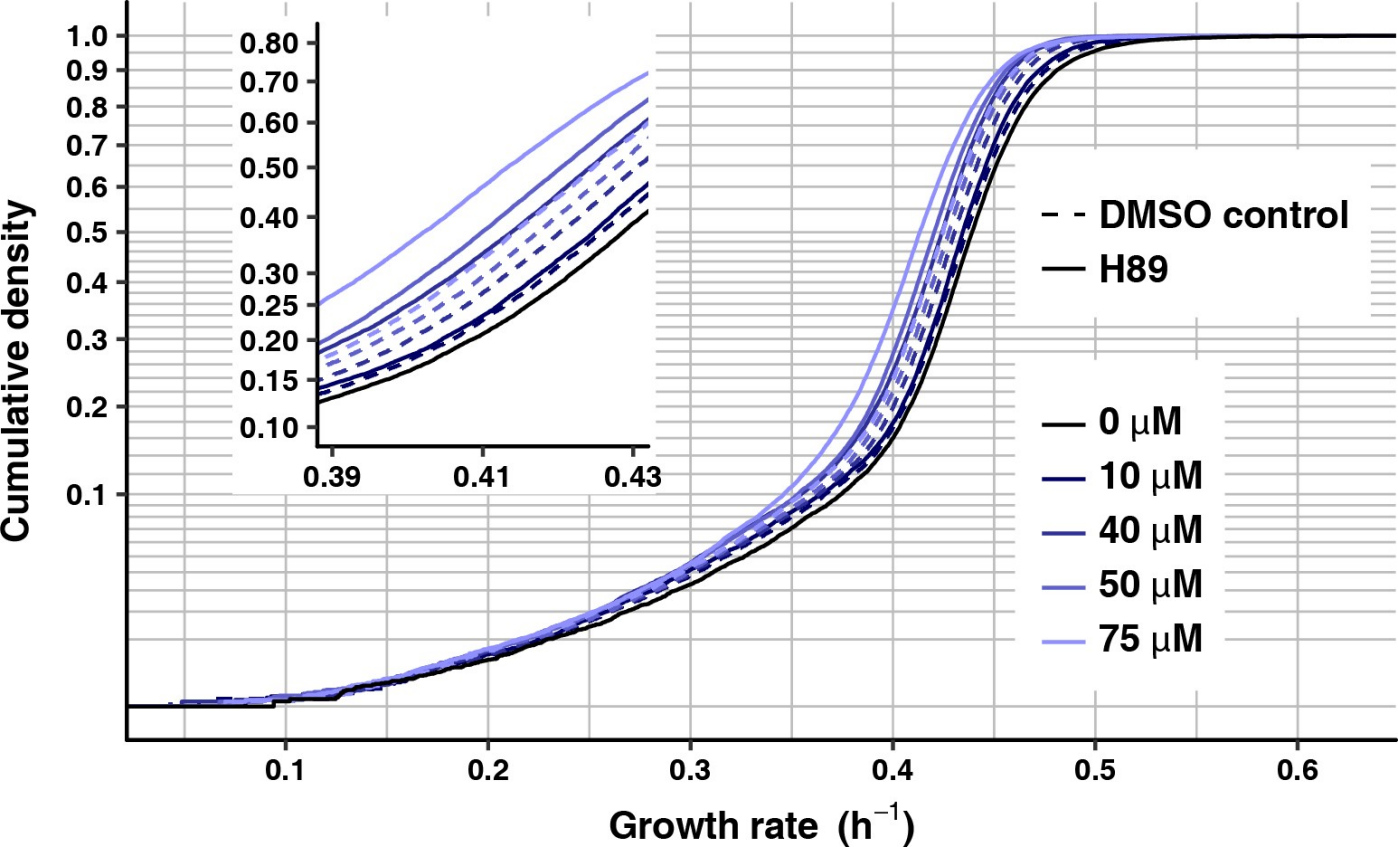
Treatment with PKA inhibitor H89 reduces growth rate. Growth-rate cumulative density curves of FY4 without H89 treatment (solid, black line, 16720 microcolonies) or treated with 10 μM (17612 microcolonies), 40 μM (19498 microcolonies), 50 μM (19528 microcolonies) and 75 μM H89 (20505 microcolonies) (solid lines of increasingly light shades of blue) and matched DMSO controls (10 μM, 16559 microcolonies; 40 μM, 19334 microcolonies; 50 μM, 18869 microcolonies; and 75 μM H89, 19551 microcolonies) (dashed lines). The inset plot shows the same data, zoomed into the growth-rate range between 0.39 and 0.43. Vertical axes are on a square-root scale for a better view of the slower-growing tail of each distribution.

Specifically, we treated cells with H89, which inhibits PKA activity in a concentration-dependent manner in yeast [97]. We cultured cells for these experiments without H89 and only exposed cells to H89 at the beginning of growth-rate monitoring, so as to assay the short-term effect of PKA impairment rather than a physiologically adapted cell state. Because H89 needs to be dissolved in DMSO, we used a separate control for each H89 concentration, which contained the same amount of DMSO but no H89. Consistent with our original prediction, populations of FY4 cells treated with a range of H89 concentrations all show mean growth rates shifted towards slow growth relative to corresponding DMSO controls (at 10 μM H89: 0.425 h^–1^, DMSO control: 0.427 h^–1^, Wilcoxon-Mann-Whitney test, *P* = 1.5 X 10^−7^; at 40 μM H89: 0.413 h^–1^, DMSO control: 0.420 h^–1^, Wilcoxon-Mann-Whitney test, *P* < 2.2 X 10^−16^; at 50 μM H89: 0.410 h^–1^, DMSO control: 0.417 h^–1^, Wilcoxon-Mann-Whitney test, *P* < 2.2 X 10^−16^; at 75 μM H89: 0.405 h^–1^, DMSO control: 0.415 h^–1^, Wilcoxon-Mann-Whitney test, *P* < 2.2 X 10^−16^) (Fig 7). The importance of the matched DMSO controls is evident, in that DMSO, even at the lowest concentration (0.1%, corresponding to 10 μM H89) has an effect on growth rate mean (no DMSO: 0.432 h^–1^, Wilcoxon-Mann-Whitney test, *P* < 2.2 X 10^−16^ for each comparison with a DMSO control).

To confirm that H89 affects the most relevant PKA target, Msn2, we assayed H89-treated and DMSO control cells for their relative Msn2 nuclear abundance, calculated as above as the ratio of median nuclear to median cytoplasmic Msn2-mRuby2 fluorescence intensity. DMSO alone induces significantly greater relative nuclear abundance of Msn2, consistent with DMSO inducing a stress response and reducing growth. At 0.75% DMSO (control for 75 μM H89), the mean relative Msn2 nuclear abundance is 1.073, relative to 1.051 for no DMSO (Wilcoxon-Mann-Whitney test, *P* < 2.2 X 10^−16^). Nonetheless, consistent with prediction, H89 yields a significant additional increase in mean relative Msn2 nuclear abundance (1.077, Wilcoxon-Mann-Whitney test, *P* = 5.9 X 10^−5^ for comparison with DMSO control) (S6 Fig).

### Increasing intracellular cAMP levels or removing Msn2 and Msn4 severely reduces tolerance of acute heat stress whereas decreased PKA activity increases tolerance of acute heat stress

To be true, our hypothesis that variability in the Ras/cAMP/PKA pathway underlies a bet-hedging strategy in budding yeast requires that manipulations of cAMP levels or of PKA or its downstream transcription factors not only alter growth and gene expression, but also alter acute stress tolerance in a consistent way. In particular, we expected increased cAMP levels or *msn2* deletion to reduce acute stress tolerance. Because *msn4* is not required for heterogeneous Tsl1 expression, *msn4* deletion might not have an effect on acute stress tolerance, or it might if stress-protective targets besides *TSL1* require Msn4. We further expected PKA impairment to increase acute stress tolerance.

**Fig 8:**
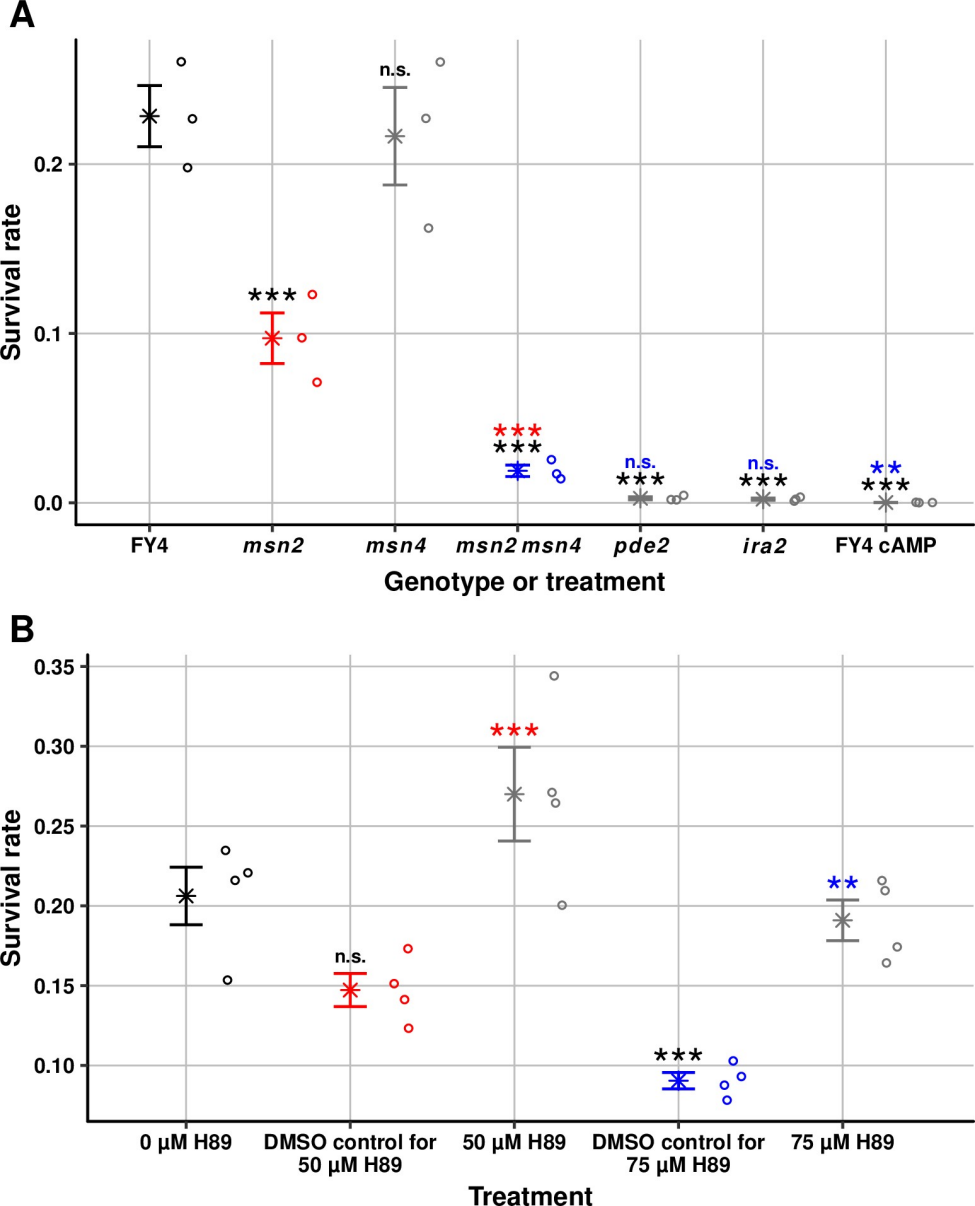
Perturbations of the Ras/cAMP/PKA/Msn2/4 pathway affect acute heat-stress tolerance. (A) Rate of survival of acute heat shock (51° C for 2 min) for FY4, *msn2*, *msn4*, *msn2 msn4*, *pde2*, *ira2* and FY4 treated with 15 mM 8-bromo cAMP. Star symbols indicate the mean. Error bars indicate standard error of the mean. Individual data points (three replicates per strain) are shown to the right of each mean-and-error bar. Statistical significance is indicated for pairwise comparisons (*** = adjusted *P* < 0.001; ** = adjusted *P* < 0.01; n.s. = not significant). Each significance notation is color coded by the genotype to which the comparison is made (and the mean-and-error bar and data points corresponding to that genotype are in the same color to guide the eye): comparisons to FY4 are in black, comparison to *msn2* is in red, and comparison to *msn2 msn4* is in blue. (B) Rate of survival of acute heat shock (51° C for 2 min) for FY4 and FY4 treated with 50 μM and 75 μM H89 and matched DMSO controls. Star symbols indicate the mean. Error bars indicate standard error of the mean. Individual data points (four replicates per strain) are shown to the right of each mean-and-error bar. Statistical significance is indicated for pairwise comparisons (*** = adjusted *P* < 0.001; ** = adjusted *P* < 0.01; n.s. = not significant). As in part A, each significance notation is color coded by the treatment to which the comparison is made and the mean-and-error bar and data points corresponding to that treatment are in the same color: comparisons to 0 μM H89 are in black, comparison to DMSO control for 50 μM H89 is in red, and comparison to DMSO control for 75 μM H89 is in blue.

To test acute stress tolerance, we first subjected populations of FY4, FY4 cultured with 8-bromo-cAMP, and *pde2*, *ira2*, *msn2*, *msn4*, and *msn2 msn4* mutants to an intense heat shock (51° C for 2 min) that produces high mortality (>75%) in the FY4 wild type (Fig 8A). The *pde2* and *ira2* mutants both show a significant reduction in survival rate of approximately 100-fold relative to FY4 (one-way ANOVA on arcsine-transformed survival rates followed by Tukey’s HSD test, adjusted *P* = 3.0 X 10^−9^ for FY4 vs. *pde2* and adjusted *P* = 2.5 X 10^−9^ for FY4 vs. *ira2*). Increasing intracellular cAMP levels through addition of 8-bromo-cAMP also significantly reduces survival, to a level that is 1000-fold lower than that of FY4 (adjusted *P* = 1.0 X 10^−9^). *MSN2* and *MSN4* appear to be partially redundant with respect to tolerance of acute heat stress, with *MSN2* being more important. The *msn2* mutant shows a significant reduction in survival rate of a little more than two-fold relative to FY4 (adjusted *P* = 1.8 X 10^−4^), whereas the *msn4* mutant shows a slight and nonsignificant reduction relative to FY4 (adjusted *P* = 1.0). Consistent with partial redundancy, the *msn2 msn4* double mutant shows a greater reduction in survival rate than *msn2* alone, of approximately 10-fold relative to FY4 (adjusted *P* = 5.3 X 10^−8^ for comparison with FY4, adjusted *P* = 2.3 X 10^−4^ for comparison with *msn2*). The results for *msn2* and *msn4* suggest that, of the genes that contribute to acute heat-stress tolerance, few if any require Msn4, some require Msn2 (such as *TSL1*), and others require neither specifically but at least one of the two. The *pde2* and *ira2* survival rates are not significantly different from that of the *msn2 msn4* double mutant (adjusted *P* = 0.082 and 0.059, respectively), but the 8-bromo-cAMP survival rate is (adjusted *P* = 6.8 X 10^−3^), which suggests that cAMP might not reduce acute-stress tolerance solely through Msn2 and Msn4.

To test the effect of PKA impairment, acute heat shocks were performed on H89-treated cells. These experiments were done separately from the heat-shock experiments above because of the need for DMSO controls for each concentration of H89. Consistent with the results that H89-treated cells have reduced mean growth rates and increased mean Msn2 nuclear occupancy, cell populations treated with 50 μM or 75 μM H89 both have significantly increased survival rates compared with their respective DMSO controls (50 μM H89: adjusted *P* = 1.0 X 10^−3^; 75 μM H89: adjusted *P* = 1.4 X 10^−3^) (Fig 8B). It is important to note that, although DMSO decreases mean growth rate and increases mean Msn2 nuclear occupancy, DMSO decreases survival rates relative to no DMSO (Fig 8B). The decreased survival rate is not statistically significant for 0.5% DMSO (adjusted *P* = 0.12) but is significant for 0.75% DMSO (adjusted *P* = 4.4 X 10^−4^). These decreases suggest that reducing mean growth rate and increasing mean Msn2 nuclear occupancy are not sufficient to confer greater stress tolerance, but instead suggest that DMSO is acting as a stress to which cells respond but succumb. The observation that H89 reverses the decreases in survival rate suggests that H89 is not merely further taxing the cells but is instead increasing their preparedness for acute stress.

### Msn2 links tolerance of acute heat stress and growth in individual microcolonies

To test how the relationship between growth and acute stress tolerance at the single-cell level is altered by loss of Msn2, we performed microcolony growth assays in which an acute heat stress (5 min at 70° C with final plate temperature measured to be 51° C) was delivered 5 h into the experiment and then heat-shocked microcolonies were monitored for an additional 15 h to assay survival (see Methods). We had previously used a similar experiment to show that Tsl1 expression correlates both with growth and with acute-stress tolerance at the single-cell level, as it does at the population level [3]. In the current experiments, we also included the *TSL1-GFP* reporter, both to confirm the prior results and to investigate whether the attenuated Tsl1 expression differences that remain after *msn2* deletion still correlate with stress tolerance. In addition to comparing wild-type *TSL1-GFP* to *msn2*-mutant *TSL1-GFP*, we also compared these genotypes to *msn4*-mutant *TSL1-GFP*, because *msn4*-mutant cells have an altered growth-rate distribution but still show a correlation between Tsl1 expression and growth.

**Fig 9:**
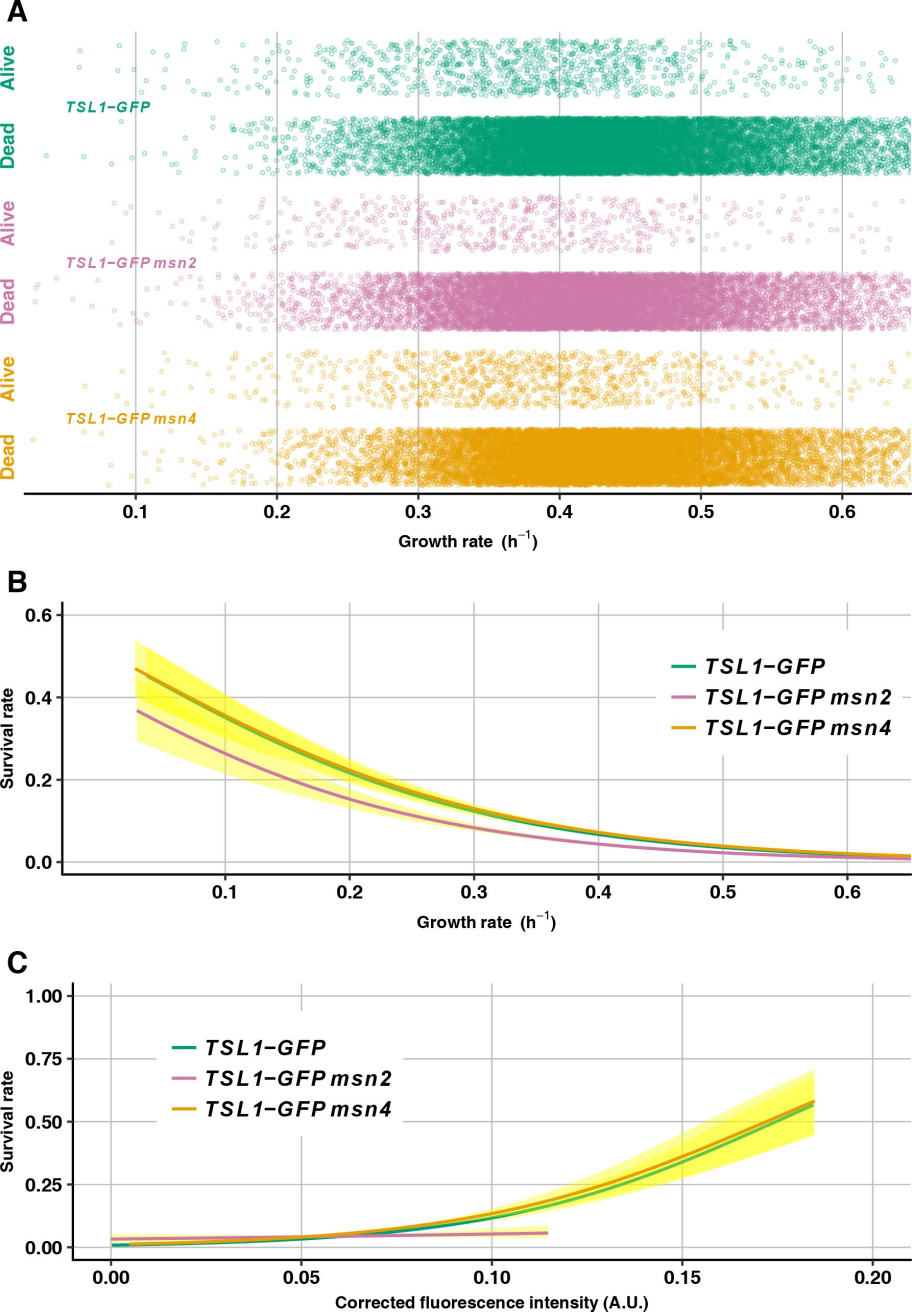
Loss of Msn2 but not Msn4 reduces stress tolerance of slower-growing cells. (A) Growth rate before heat shock is plotted for microcolonies that were alive or dead after heat shock for the genotypes *TSL1-GFP* (green, 16528 microcolonies), *TSL1-GFP msn2* (light purple, 14123 microcolonies) and *TSL1-GFP msn4* (light orange, 15683 microcolonies). Points are jittered vertically to better see them. (B) Logistic regression of survival rate on microcolony growth rate for *TSL1-GFP* (green), *TSL1-GFP msn2* (light purple) and *TSL1-GFP msn4* (light orange), with 95% confidence intervals shown in yellow. (C) Logistic regression of survival rate on mean GFP fluorescence intensity (corrected as in Fig 6) for *TSL1-GFP* (green), *TSL1-GFP msn2* (light purple) and *TSL1-GFP msn4* (light orange), with 95% confidence intervals shown in yellow.

The wild-type *TSL1-GFP* strain does indeed recapitulate prior results, in that individual microcolonies are more likely to survive the slower they are growing before heat shock (Fig 9A and 9B) and the more they are expressing Tsl1 before heat shock (Fig 9C). Deletion of *msn4* has negligible effect on survival rate, consistent with the results above from the heat shocks in bulk populations (Fig 9A and 9B). In contrast, deletion of *msn2* reduces the survival rate, and this reduction is most pronounced at slow growth rates (Fig 9A and 9B). In other words, those slower-growing cells that exist despite *msn2* deletion have reduced stress tolerance compared with equally slow-growing cells that exist in the wild type. That said, the relationship between slow growth and survival is not completely eliminated by *msn2* deletion (Fig 9B), suggesting that there is a residual, Msn2-independent mechanism that ties slow growth to stress tolerance. As shown in Fig 9C, *msn2* deletion eliminates the correlation between Tsl1-GFP expression level and survival (whereas, as expected, *msn4* deletion does not). One possible explanation for this result relates to the finding of altered covariances of Msn2 targets upon gene-dosage perturbations of Ras/cAMP/PKA pathway members [53]. Specifically, it could be that the cell-to-cell differences in Tsl1 expression that remain after *msn2* deletion are not correlated with cell-to-cell differences in other Msn2-dependent stress-tolerance factors, as they would be in wild-type cells, so the total amount of stress protection averages out.

## Discussion

**Fig 10:**
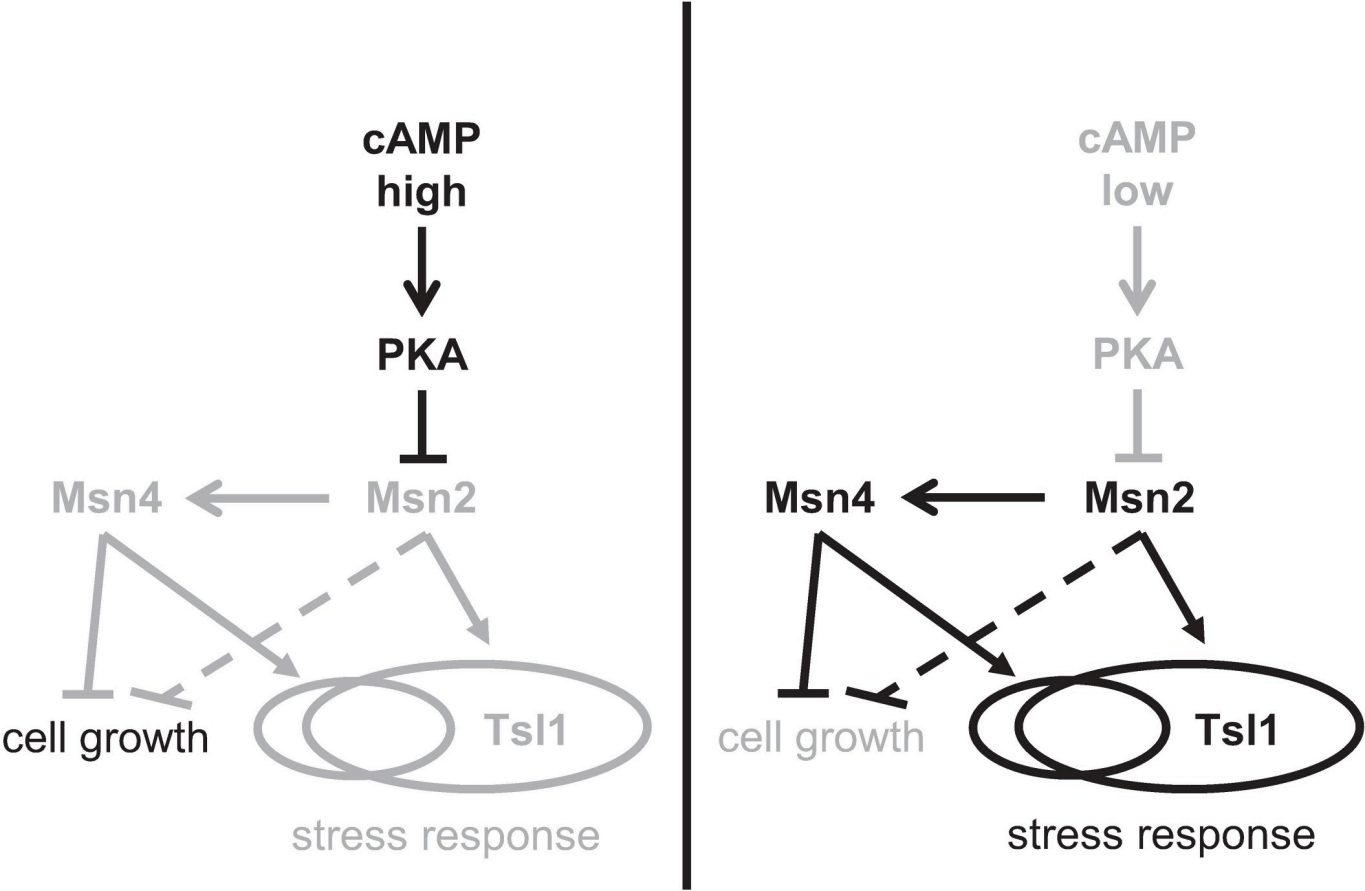
Model of role of cAMP/PKA/Msn2/4 pathway in regulating nongenetic heterogeneity of growth rate and stress tolerance. cAMP levels differ between isogenic cells under benign conditions. When intracellular cAMP concentration is high in a cell (left panel), PKA activity is increased, which in turn suppresses the activity of Msn2 and hence Msn4. Msn2 and Msn4 therefore do not suppress cell growth or activate their target stress-response genes, leading to a faster-growing, stress-susceptible state. Because Msn2 induces Msn4, it is possible that Msn2 does not affect growth-rate heterogeneity directly (dashed T-bar arrow) but instead acts through Msn4. When intracellular cAMP concentration is low in a cell (right panel), PKA activity is reduced, which in turn leads to higher nuclear occupancy of Msn2, leading to higher activity of Msn4 as well. Msn2 and Msn4 suppress cell growth and activate their partially overlapping sets of target stress-response genes, such as *TSL1* (which solely requires Msn2), leading to a slower-growing, stress-tolerant state. Black arrows and text denote high activity or abundance, whereas grey arrows and text denote low activity or abundance.

The results of this study are consistent with a model (Fig 10) in which cAMP levels differ in isogenic cells cultured in the same, benign environment, leading to cellular heterogeneity in the activities of the transcription factors Msn2 and Msn4 (in particular their nuclear occupancy) and thereby to cellular heterogeneity in growth rate and stress tolerance. Mutations that increase intracellular cAMP levels (*ira2* and *pde2* deletions) or remove the transcription factors (*msn2* and *msn4* deletions) reduce growth heterogeneity by reducing the abundance of slower-growing cells, as does culturing of wild-type cells with the cell-permeable cAMP analog 8-bromo-cAMP. These manipulations also reduce tolerance of cells to acute heat stress, although the effect of *msn4* deletion is only seen in combination with *msn2* deletion, suggesting that heterogeneous expression of stress-tolerance genes primarily requires Msn2 (but in some cases loss of Msn2 can be compensated by functional Msn4). *TSL1* appears to be one such gene that requires Msn2 but not Msn4. Whereas *msn4* deletion has no detectable effect on Tsl1 expression heterogeneity, *pde2* or *msn2* deletion reduces Tsl1 expression heterogeneity by eliminating high-expressing cells, as does culturing of wild-type cells with 8-bromo-cAMP. Taken together, our results show that Msn2 has a major, but not exclusive, role in linking slower growth to higher tolerance of acute heat stress, and that heterogeneity in Msn2 activity is most likely caused by cell-to-cell differences in cAMP levels.

One important feature of this model is that slow growth and stress tolerance are not inevitably linked. A connection between slow growth and stress tolerance has been observed in several systems [4, 14, 31, 32], but our results argue that this connection is to some extent an evolved one rather than an automatic one. In particular, we have added to our previous finding that *tsl1* deletion does not appreciably alter the distribution of microcolony growth rates [3] the findings that: 1) Msn4 is not required for heterogeneous Tsl1 expression but is required for normal abundance of slower-growing cells, and 2) there are both Msn2-dependent and Msn2-independent slower-growing cells that have different tolerances of acute heat stress.

Two features of this model are difficult to reconcile with our experimental data, but we believe these discrepancies do not negate the general conclusion that the Ras/cAMP/PKA pathway, acting through Msn2 and Msn4, underlies nongenetic heterogeneity in yeast-cell growth and stress tolerance. First, the *pde2* deletion not only reduces the abundance of slower-growing cells in the left tail of the microcolony growth-rate distribution but also increases the abundance of cells with intermediate growth rates (the shoulder of the growth-rate distribution). We suspect that the exact nature of the *pde2* deletion matters, perhaps due to effects on neighboring genes, because of our unexpected finding that, contrary to a prior report using a different deletion, *pde2*-deleted cells can survive as petites. Further work will be necessary to untangle the effect of Pde2 on growth heterogeneity. In the meantime, we note that the clear effects of *pde2* deletion on Tsl1 expression heterogeneity and on acute heat-stress tolerance are as predicted by the model. Second, mutations in PKA components have effects that are opposite to expectations. The model predicts that removing PKA activity (by *tpk1* or *tpk2* deletion) would shift the growth-rate distribution toward slower-growing cells and that enhancing PKA activity (by *bcy1* deletion) would reduce the abundance of slower-growing cells. We can only state that this is not the first time that unexpected results have been seen with mutations in PKA components [41] and that further work will be necessary to determine how our results might be explained by PKA’s pleiotropic activities, including activities that do not go through Msn2 and Msn4, by the unclear functional overlaps between its alternate catalytic subunits, or by complex feedbacks and cross-talk with other signaling pathways. In the meantime, we have demonstrated that the immediate consequences of mild impairment of PKA activity by H89 are as predicted by our model: mean growth rate decreases, mean Msn2 nuclear occupancy increases, and acute stress tolerance increases.

The major unknown in our model is the ultimate source of differences in cAMP levels between cells. Because culturing of wild-type cells in 8-bromo-cAMP reduces heterogeneity, we infer that the source of heterogeneity is upstream of cAMP. However, this inference leaves many possibilities. As a second messenger molecule, cAMP integrates various environmental signals, including those received through sensors of glucose and nitrogen concentrations as well as sensors of oxidative stress, heat stress and DNA damage [98–108]. The DNA-damage response is the only potential source of heterogeneity that we have ruled out as being a major contributor to growth heterogeneity (although, to be clear, we have not ruled out that the small minority of the population that have been characterized as DNA damage-induced persisters [74] contributes to evolutionary adaptation). Even though we culture cells in rich medium without nominal stress, nutrients and stressors might still show microenvironmental fluctuations that could trigger cAMP changes, or the nutrient or stress sensors might have variable abundances or activities among cells. Moreover, intracellular cAMP levels are regulated by feedback loops emanating from PKA itself [109], so as is the case with all feedback-including networks, definitions of upstream and downstream break down.

Any candidate source of heterogeneity must be inherited across cell generations, because a daughter cell tends to inherit the approximate growth rate of its mother, with occasional switches from slower growth to faster growth or vice versa [3]. Chromatin-based mechanisms, which can have switching rates on the order of tens of generations [110], might be good candidates, although such mechanisms would need to generate a continuous distribution of growth states rather than a simple bimodal distribution. One theoretical mechanism that has been proposed for long-lasting persistence of any particular state along a continuum is kinetic memory, which can be achieved when a system of enzyme-catalyzed reactions includes an enzyme that is present at a concentration lower than that of its substrates [111].

We propose that one specific candidate for the source of inherited heterogeneity is cytosolic pH, which affects Ras signaling. Around neutral cytosolic pH, there is a positive relationship between cytosolic pH and growth, as the vacuolar H+-ATPase (V-ATPase) promotes growth by activating Ras, which increases intracellular cAMP concentration [112]. The major regulator of cytosolic pH is Pma1, a long-lived protein that is asymmetrically localized to the plasma membrane of mother cells. A newborn cell begins with a very low level of Pma1, derived from new synthesis; as generations progress, the Pma1 level increases in this cell [113]. Low Pma1 means low cytosolic pH, so a newborn cell has lower cytosolic pH than its mother [113]. If Pma1 expression is heterogeneous, then a subset of newborn cells might have especially low cytosolic pH that takes many generations to catch up (which could give rise to slower-growing microcolonies). Alternatively or additionally, if Pma1 asymmetric inheritance is heterogeneous, then a subset of newborn cells might have especially high cytosolic pH (which could give rise to faster-growing microcolonies). Pma1 undergoes post-transcriptional modification along cell division [114], so Pma1 in aged cells could have altered function, which might explain the observation that slower-growing cells tend to be replicatively older than faster-growing cells [3], despite having higher cytosolic pH [113]. Indeed, it has been suggested that a mechanism other than Ras activation through the V-ATPase could be involved in regulating cell growth at alkaline cytosolic pH [112].

The Ras/cAMP/PKA pathway is highly conserved across eukaryotes [82, 115, 116] so our findings might have relevance to human disease. As it does in yeast, cAMP acts as a second messenger in human cells and regulates a wide range of functions. In humans, these functions include ion-channel conductivity, synaptic release of neurotransmitters, cytoskeletal remodeling, metabolism, proliferation, differentiation and apoptosis [117, 118]. Dysregulation of cAMP levels is associated with the onset or progression of various cancers [119–124]. For example, activating the cAMP/PKA pathway is one way to tolerate glucose depletion and acquire aggressive growth in cancer cells [125]. Likewise, in a subset of melanomas, increased cAMP levels confer resistance to MAP kinase pathway inhibitors in a PKA-dependent manner, presumably by promoting growth through an alternate route [13]. However, because different cell types may regulate cAMP levels differently and show differential expression and localization of PKA subunits, cAMP can work as a growth activator or inhibitor in different cell lines [119, 122, 124, 126, 127]. As a result, for some cancers but not others, cAMP or PKA can be a very good target of therapy, and some cAMP analogs and PKA antisense oligonucleotides have been tested as anticancer agents [128–131]. Either way, to the extent that cell-to-cell differences in Ras/cAMP/PKA pathway activity exist, they could contribute to nongenetic heterogeneity in tolerance of chemotherapy within a tumor. Appropriate manipulations of cAMP levels or PKA activity might therefore reduce heterogeneity and thereby render a tumor-cell population more uniformly susceptible to a second drug.

This strategy, of manipulating cAMP levels to reduce nongenetic heterogeneity so that a second drug has higher efficacy, might also be valuable for treating fungal infections. For example, if pathogenic *S*. *cerevisiae* show the same form of nongenetic heterogeneity that the strain background we used does, then increasing cAMP levels might render an infection more susceptible to fever or another drug. Of course, care must be taken for two important reasons. First, the principle behind the strategy is that an infectious population will be made to grow faster on average than it otherwise would, because slower-growing, stress-tolerant cells are reduced. If an appropriate, effective stress is not applied concurrently, then the infection could be made worse. Second, any therapy administered to treat a fungal infection may also impact otherwise-healthy host cells. Increasing cAMP levels in infecting yeast might render the patient more susceptible to particular cancers, or less treatable should they arise. Perhaps one cautious way forward would be to examine microbiome composition of patients already being treated with cAMP-increasing drugs, such as cAMP analogs or forskolin, which activates adenylyl cyclase.

## Methods

### Yeast strains

All strains used in this study are prototrophic *MATa* haploids derived from the wild-type FY4 background, except the *HSP12-mCherry*; *RNR3-GFP* strain (BY4741, *MATa*, *his3 leu2 met15 ura3*, *RNR3-EGFP-HIS*; *HSP12-mCherry-KanMX*), which was acquired from Gilad Yaakov and Naama Barkai [74].

Unless otherwise specified, all mutants were made so as to cleanly delete genes without leaving marker or vector sequences behind. We used the *KanMX*; *pGAL-p53* (*Kp53*) *delitto perfetto* cassette [132], as follows. The entire coding sequence of a target gene was replaced with *Kp53* by homologous recombination and selected for resistance against G418, using standard protocols. Next, a construct concatenating the flanking sequences of the target gene was used to excise the entire *Kp53* cassette by homologous recombination and selection on galactose plates. Cells that retained the cassette would have p53 expression, which is toxic and represses cell growth. The *bcy1* deletion used in this study retained the *Kp53* cassette because selection against p53 was not possible as the *bcy1* mutant cannot utilize galactose.

A *TSL1-GFP* strain and an *MSN2-mRuby2*, *HTB2-GFP*, *TSL1-mTFP1* strain were also constructed using the *delitto perfetto* cassette, so that there is no marker gene accompanying insertion of fluorescent-protein coding sequence. First, the *Kp53* cassette was inserted by homologous recombination at the 3’ end of the coding sequence of the target gene, replacing its stop codon. Then, coding sequence of the fluorescent fusion protein, including sequence immediately 3’ of the stop codon of the target gene, was transformed and selected on galactose to remove the *Kp53* cassette and introduce the fusion to fluorescent-protein coding sequence. The transformation construct for this step was either PCR-amplified from the GFP-fusion sequence that existed (alongside an auxotrophic marker) in the yeast GFP collection used previously [3] or made by overlapping PCR to paste together coding sequences of the 3’ end of the target gene, a linker, and the fluorescent protein.

The double-mutant *msn2 msn4* strain was made by crossing the *msn2* and *msn4* single mutants. *TSL1-GFP msn2*, *TSL1-GFP msn4*, *TSL1-GFP msn2 msn4*, and *TSL1-GFP pde2* strains were made by crossing the respective single-mutant or double-mutant strains with the *TSL1-GFP* strain. The *MSN2-mRuby2*, *HTB2-GFP*, *TSL1-mTFP1* strain was made by crossing the *MSN2-mRuby2*, *HTB2-GFP* and *HTB2-GFP*, *TSL1-mTFP1* strains. In these crosses, tetrads derived from the F1 diploids were tested for genotype and mating type with colony PCR and halo assays, respectively, using standard methods.

For all generated strains, several (typically three) independent biological clones were isolated. Preliminary validation experiments, with replicates performed on different dates, were done to ensure clones did not differ in their microcolony growth-rate distributions and fluorescent-reporter expression, then a single clone per genotype was chosen for subsequent experiments to maximize throughput.

### Media and strain cultivation

For all strains, -80° C frozen stocks were made from saturated YPD liquid cultures. Two days prior to any microscopy experiment, strains from the frozen stocks were inoculated into Synthetic Complete (SC) liquid medium and grown to saturation overnight. For the *ira2* mutant, a larger inoculum was used to compensate for its slower average growth rate. Because the *bcy1* deletion mutant cannot utilize ethanol as a carbon source, saturation was intentionally avoided by using a small inoculum. One day before a microscopy experiment, saturated cell cultures were diluted into fresh SC liquid media by a factor of 2X10^5^, to about 1X10^3^ cells/ml, except in the cases of *ira2* and *bcy1* mutants, for which the diluted concentration was about 2X10^4^ cells/ml. For addition of the cAMP analog, fresh SC liquid medium was supplemented with 15 mM 8-bromo-cAMP (Sigma, Catalog Number: B7880, dissolved in water) and culture tubes were wrapped with foil to protect the light-sensitive compound. *ira2* and *bcy1* mutants were diluted to about 2X10^4^ cells/ml into fresh SC liquid media. After dilution, all strains were cultivated for 18 h in log phase before the experiment to ensure that all cells had exited lag phase and to ensure that the cAMP analog had an opportunity to have an effect. For treatment with H89 (Sigma, Catalog Number: B1427, dissolved in DMSO), cells were cultivated for 18 h in log phase in the absence of H89, then transferred to medium with H89 at the onset of growth-rate monitoring, so as to test the immediate effect of PKA perturbation rather than the effect of physiological adaptation to low PKA activity. For all strains, cell densities before microscopy were approximately 10^6^ cells/ml.

### MitoTracker staining

Cells were stained with MitoTracker Red CMXRos (ThermoFisher, Catalog Number: M7512) for petite exclusion. Staining was performed immediately before microscope plating, at approximate cell densities of 10^6^ cells/ml. For each strain, 200 μL of cell culture was aspirated into a 1.5-ml opaque black tube, which protects the staining reaction from light. After a brief centrifugation at 2320 x *g* for 1 min, 100 μL of supernatant was removed, leaving 100 μL behind. In this way, cells remained immersed in the same culture media during staining. We found this step to be crucial for successful staining. MitoTracker stains cells based on mitochondrial inner membrane potential, which is directly related to respiration activity. The 10^6^ cells/ml stage is good for such staining, as it ensures that cells are still in log phase, but also ensures that normal cells should already have weak respiration while petites should have none. Using water instead of the resident media would cause a change in glucose concentration, and hence respiration activity, depending on the genotype. Using fresh SC instead of the resident media would cause an increase in glucose that could suppress any respiration, making normal cells look like petites. Using a consistent cell density (10^6^ cells/ml) kept glucose levels consistent across genotypes.

To prepare the MitoTracker dye for each experiment, 50 μg was dissolved in 50 μL of DMSO and then diluted with 1 ml of sterile water. Then 10 μL of this solution was added into each tube and mixed well with cells and remaining media. The dye/cell mixture was then kept at room temperature for 10 min. To terminate staining, the dye was diluted by addition of 1 ml of fresh SC, cells were immediately centrifuged at 2320 x *g* for 1 min, and supernatant was removed as quickly and as completely as possible. Then cells were resuspended in 1 ml fresh SC. Adding fresh SC at this point does not cause problems because export of the dye, once imported, is slow.

For cells growing on YPD agar plates, petite staining was performed with 2,3,5-triphenyltetrazolium chloride (TTC) staining. Colonies were grown on YPD agar plates for at least two days before overlaying with TTC [133]. TTC, originally white, is reduced to a red form in normal cells by various dehydrogenases within functional mitochondria, whereas in petites it remains white. TTC-overlaid plates were incubated at 30° C in the dark for 30 min while the agar solidified and the TTC stain developed. Then colonies on each plate were manually counted based on color.

### High-throughput microscopy growth rate and fluorescence assay with petite control

The microcolony growth assay was performed as in previous work [3] with some modifications. In brief, 96-well glass bottom plates were coated with concanavalin A (Type IV, Sigma, Catalog Number C2010-1G) then washed with water and filled with 390 μL of fresh filtered SC per well. Prepared cells were counted with a hemocytometer and diluted to approximately 4X10^5^ cells/ml. Unlike in the original protocol [3], cells were not sonicated prior to plating, as this step was unnecessary to separate cells and could introduce a stress. Into each well, 10 μL of cell culture was seeded, making the final density approximately 4000 cells per well. For cells treated with 8-bromo-cAMP, the compound was added to a final concentration of 15 mM in a dark room after seeding the plate, to minimize exposure to light. For cells treated with H89 or DMSO, appropriate volumes were added to the SC medium in each well before seeding cells. Plates were sealed with a breathable plate membrane film (Sigma, Catalog Number Z380059-1PAK) that permits air exchange and prevents condensation, and were then spun at 453 x *g* for 2 min to help cells settle onto the glass surface.

For time-lapse analysis of microcolony growth, all images were captured on a Nikon Eclipse Ti inverted microscope with Nikon plan Apo 10X air objective and 1.5X magnification, once per hour for 10 h. For experiments in which Tsl1-GFP signal was monitored, a well mask was used to space out image fields in each well to avoid optical stress imposed by repeated short-wavelength light exposure when imaging neighboring fields. Similarly, a well mask was used for experiments with cells cultured with 8-bromo-cAMP, to reduce loss of active 8-bromo-cAMP by repeated light exposure.

The growth rates of microcolonies were measured by tracking their areas at each time point [3, 6]. We developed and used a new algorithm, PIE, for detecting microcolony edges using changes in direction of the pixel-intensity gradient [134]. PIE improves robustness against variations in focal plane and brightness and thereby achieves lower standard errors of growth-rate estimates [134].

As in prior work, growth-rate data were analyzed with linear mixed-effect modeling to partition random effects from imaging field, well, and, when relevant, replicate plate and the interaction between strain and plate [6]. Four replicate experiments from different dates were used to assay Msn2 subcellular localization dynamics (Fig 5B), as the necessary 1-min imaging interval heavily limits sample size. Two replicate experiments from different dates were used to assay microcolony growth rates across the range of H89 concentrations (Fig 7) because preliminary experiments indicated that mean differences between adjacent concentrations were small. Two replicate experiments from different FACS run dates were used to assay microcolony growth rates of founder cells from different bins of Rnr3-GFP fluorescence intensity (S1D Fig) because preliminary experiments indicated that mean differences between adjacent bins were small.

For microcolony-growth experiments recording fluorescence from MitoTracker staining and/or Tsl1-GFP, fluorescent images were only acquired at the first time point, to reduce phototoxicity. Fluorescence intensity of each cell was calculated as the median intensity of the cell body minus the median intensity of the immediate background within the smallest rectangle bounding the cell. Because cells with extremely low or no signal could then have negative intensities, each value was then further corrected by subtracting the minimal value of the entire experiment. Estimates of petite frequency for validating the MitoTracker staining approach came from the six microcolony-growth experiments conducted on the FY4 genotype.

Partitioning around medoids was conducted in R with the cluster package, using Euclidean distance and standardized growth-rate and fluorescence values to weight these two dimensions equally.

### Fluorescence-Activated Cell Sorting (FACS)

For FACS, strains were prepared in the same way as for microscopy experiments, with one exception: during the inoculation one day before FACS, an additional culture tube for *TSL1-GFP* was prepared with half the regular inoculation concentration. The purpose of this extra tube was to enable an extended FACS session. The more dilute culture replaced the regular one after two hours of sorting, which keeps the cell density around the same over this extended period. All samples were first gated by the ratio of Forward Scattered Area (FSC-A) to Forward Scattered Width (FSC-W) to select for single cells, which avoids the noise on the high-intensity end caused by cell clumps. The *TSL1-GFP* strain was sorted based on GFP intensity. Two bins of cells, the top 10% and bottom 50%, were collected into separate 5-ml tubes for further analysis. For each sort, ~5X10^4^ cells from each group were set aside for growth-rate assays, which were used to assess the quality of the sort by measuring the enrichment of slower-growing cells in the top 10% bin compared with the bottom 50% bin. During sorting, cells and the culture media are mixed with sheath liquid (PBS) to form a fine stream, which depending on the mixture proportions could lead to a non-negligible drop in glucose concentration. Yeast intracellular cAMP levels respond to extracellular glucose concentration changes at the time scale of minutes [135]. However, the fact that the growth-rate distribution of the top 10% bin still shows a significant shift toward slower growth indicates that our separations are not greatly impacted by this temporary mixing with PBS.

The *HSP12-mCherry*; *RNR3-GFP* strain was prepared in the same way as for microscopy experiments. From each desired bin of GFP intensity, as well as from the ungated population, 7X10^4^ cells were collected and then plated for growth-rate and Hsp12-mCherry fluorescence-intensity measurements.

The *MSN2-mRuby2*, *HTB2-GFP*, *TSL1-mTFP1* strain was prepared in the same way as for microscopy experiments. 3X10^4^ cells in the top 0.2% bin of Tsl1-mTFP1 signal were collected for microcolony assays to measure Msn2 subcellular localization and growth rate.

### cAMP quantification and analysis

To measure cAMP levels, FACS-sorted cells were pelleted with mild centrifugation at 2320 x *g* to remove PBS, then resuspended in fresh SC at 5X10^7^ cells/ml and allowed to recover for 30 min at 30° C. After the recovery, 20 μL of each cell culture was set aside for growth-rate assays and 0.5 μL was set aside for petite-frequency counting. The remaining cell culture was pelleted at 15682 x *g* for 30 sec, and pellets were immediately flash frozen in liquid nitrogen. Cells can be stored at -80° C for days after this step. Cells from different FACS sessions were pooled together to extract enough cAMP for ELISA detection. During pooling, the top 10% and bottom 50% bins from the same session remained paired. Three pairs of top 10%/bottom 50% samples were measured for cAMP concentration, with three technical replicates per sample.

An ELISA-based assay was used to quantify intracellular cAMP. On the day of the ELISA assay, 0.1 M room temperature HCl was used to resuspend cell pellets with a quick thaw. The appropriate volume of HCl was calculated based on cell number estimates from the growth-rate assay for each sample, to ensure consistency among samples in the final cell concentration as well as to ensure high precision of the denominator when estimating the cAMP concentration per cell. Resuspended cells were immediately vortexed at top speed at 4° C for 30 min in Macherey-Nagel Nucleospin tubes containing a 1:1 mixture of type B and C beads (Catalog Number, Type B: 740812.50; Type C: 740813.50). This vortexing physically shreds yeast cells to release cell contents including cAMP. HCl deactivates any phosphodiesterases upon shredding, protecting released cAMP from catabolism. After vortexing, tubes were centrifuged at 15682 x *g* for 5 min and the supernatants passed through Millex Millipore 0.22 μm PVDF syringe filters to remove any glass debris that could not be pelleted. The filtered supernatants were used for cAMP quantification using the Enzo Direct ELISA kit (Catalog number ADI-900-066). The lme4 package in R was used to perform linear mixed-effect modeling to separate the random noise caused by biological and technical replicates from the real difference between Tsl1-high and Tsl1-low fractions [136].

### Msn2 intracellular localization imaging

To image Msn2 intracellular localization, images were captured using a 40X air objective and 1.5X magnification with a well mask to space out imaging fields and thereby reduce phototoxicity and photobleaching in neighboring fields. Bright-field, TXRed and GFP channels were captured. For H89 treatment, images were only collected at one time point. For measurement of Msn2 subcellular localization, images were taken at 1-min intervals for 30 min, with exposure times of 5 ms for bright-field, 500 ms for TXRed, and 2 ms for GFP. Immediately following these 30 min, a 10-h microcolony growth-rate assay was performed with only bright-field imaging at 1-h intervals.

Bright-field images were processed with the same microcolony-recognition algorithm used in the growth-rate analysis to produce a binary mask identifying pixels within or outside of cells and a bounding-box binary mask identifying pixels within or outside the smallest rectangle containing each cell. Fluorescent images from the TXRed and GFP channels were imported into CellProfiler [137] along with the cell mask to segment each cell into the nucleus and the cytoplasm. Nuclear area was detected from the Htb2-GFP fluorescent signal by the Global Otsu threshold method with the nuclear diameter restricted to lie between 10 and 30 pixels. For colonies with more than one cell, such as a mother-daughter pair, individual cells were segmented by the Propagation method, which uses the number and relative positions of nuclei and the shape information from the cell mask. Full details are provided in the implemented CellProfiler project (S1 File), and the data from each cell are provided in S2 File and S3 File. Msn2-mRuby2 pixel intensities were then recorded for each compartment in each cell. The relative Msn2 nuclear abundance for H89 treatment was calculated as the ratio of the median intensity in the nucleus to the median intensity in the cytoplasm (S2 File). For measurement of Msn2 subcellular localization, it was necessary to include an additional background correction, because Msn2 signal under benign conditions is not as strong. This background correction was implemented before taking the ratio by subtracting from each median intensity the median intensity within the bounding box but outside the cell (S3 File). For both H89 treatment and Msn2 subcellular localization, to confirm robustness of conclusions drawn, ratios were also calculated using mean intensities or upper-quartile intensities, rather than median intensities; conclusions did not differ so only the ratios using the median are reported.

For the experiments linking Msn2 subcellular localization dynamics with microcolony growth, the cells analyzed by CellProfiler for Msn2 tracking were connected to microcolonies by their centroid coordinates.

### Acute heat shock

To assay survival of acute heat stress, cells were prepared the same way as for growth-rate assays. For each strain, cell density was estimated by counting cells with a hemocytometer. Based on preliminary experiments, some strains were found to have very low survival so more cells of these strains were assayed per experiment. For the FY4, *msn2*, *msn4*, and *msn2 msn4* strains and DMSO- or H89-treated FY4, cells were diluted in fresh SC to 2X10^4^ cells/ml. For the *pde2* and *ira2* strains, and for FY4 cultured with 8-bromo-cAMP, cells were diluted in fresh SC to 2X10^5^ cells/ml.

For each strain, the diluted cell culture was mixed well and 100 μL was immediately aliquoted into each of three or four control tubes. After remixing within each control tube, 50 μL of each was aspirated into a matched heat-shock tube. All tubes were 0.2-mL thin-walled tubes usually used for PCR reactions. The three or four heat-shock tubes for each strain were heat shocked for 2 min at 51° C in a thermal cycler with heated lid. Samples were then quickly plated individually on YPD plates. Appropriately sized aliquots of each sample (based on preliminary data on survival rates so that surviving colonies were neither too sparse nor too crowded) were plated on individual YPD plates and incubated at 30° C for two days. TTC agar was then overlaid to identify petites, and non-petite colonies were used to calculate survival rates (as a ratio of number of colonies surviving heat shock to number of colonies of matched control, multiplied by the appropriate dilution factor). Twenty such plates without heat-shock treatment were used to estimate FY4 petite frequency by direct scoring.

### Acute heat shock of microcolonies

Heat shocks of microplates were performed similarly to those in our previous study [3] with minor changes. Preliminary experiments indicated that heating of wells on the perimeter of the plate was inconsistent with heating of wells on the interior, so none of the perimeter wells were used. Microplates were heated by resting them in preheated stainless steel shot and covering with a preheated aluminum block. Preheating was conducted in an incubator set to 70° C, as were heat shocks, which lasted 5 min, a duration we determined to produce a final temperature of 51° C within wells. Growth of microcolonies for 5 h before heat shock and for 15 h after heat shock was tracked as above, with the two phases of the experiment processed separately. Microcolonies from the two phases were connected subsequently by their centroid coordinates in a way that accommodated a global shift in coordinates caused by removing the microplate from the microscope and returning it. A microcolony was considered dead if its area did not increase by at least two-fold during the 15 h after heat shock.

## Supporting information

S1 Fig DNA damage does not contribute substantially to the slow-growing cell population. (A) Mean Hsp12-mCherry fluorescence intensity—corrected by subtracting local background fluorescence then by subtracting the minimum value for the entire experiment, to avoid negative values (see Methods, vertical axis)—is plotted against microcolony growth rate (horizontal axis). Total sample size is 59183 microcolonies. The solid line is the fit to a generalized additive model with cubic spline smoother, with 95% confidence interval shown in yellow. Vertical axis is on a square-root scale for a better view at the low-intensity end. (B) Quantile-quantile plot of fluorescence intensity of Rnr3-GFP strain versus no-GFP control. For each strain 12978 cells were recorded. The dashed grey line has intercept 0 and slope 1; the solid grey line is the least-squares linear best fit of the quantile-quantile plot between the bottom 10% and 25% quantiles. Points highlighted in magenta correspond to the region between the top 20% and 25% quantiles. (C) Growth-rate cumulative density curves of FACS-gated top 0.2% *RNR3-GFP* cells (red, 4556 microcolonies) and ungated cells (black, 59183 microcolonies). Vertical axis is on a square-root scale for a better view of the slower-growing tail of each distribution. (D) Growth-rate cumulative density curves of the following FACS-gated bins of *RNR3-GFP* cells with 0% being the most intense: 0–2% (43393 microcolonies), 5–7% (44201 microcolonies), 10–12% (41465 microcolonies), 20–25% (37048 microcolonies) (shown in increasingly light shades of red), and ungated cells (black, 39617 microcolonies). Vertical axis is on a square-root scale for a better view of the slower-growing tail of each distribution.
(TIF)

S2 Fig Intracellular cAMP controls nongenetic heterogeneity in Tsl1 expression. Same data as in Fig 4B plotted in separate panels for each genotype or treatment. Mean GFP fluorescence intensity—corrected by subtracting local background fluorescence then by subtracting the minimum value for the entire experiment, to avoid negative values (see Methods, vertical axis)—is plotted against microcolony growth rate (horizontal axis) for (A) FY4 no-GFP control (black, 7340 microcolonies), (B) *TSL1-GFP* (green, 6912 microcolonies), (C) *TSL1-GFP* cultivated with 15 mM 8-bromo-cAMP (orange, 3730 microcolonies) and (D) *TSL1-GFP pde2* (blue, 1778 microcolonies). Each solid line is the fit to a generalized additive model with cubic spline smoother, with 95% confidence interval shown in yellow. Vertical axis is on a square-root scale for a better view at the low-intensity end.
(TIF)

S3 Fig Petites not filtered by MitoTracker staining do not explain correlation between Tsl1 abundance and growth rate. (A) Same plot of *TSL1-GFP* data as in Fig 4B, with two clusters identified by partitioning around medoids indicated in black (higher growth rate, lower Tsl1 abundance) and green (lower growth rate, higher Tsl1 abundance). (B) Same plot of *TSL1-GFP* data as in Fig 4B, with microcolonies color coded by MitoTracker staining (black = lowest 3% of MitoTracker staining of microcolonies that passed the MitoTracker-staining threshold, red = highest 97% of microcolonies that passed the MitoTracker staining) and with additional data shown for microcolonies that had not passed the MitoTracker-staining threshold (grey).
(TIF)

S4 Fig Msn2 but not Msn4 is required for nongenetic heterogeneity in Tsl1 expression. Same data as in Fig 6B plotted in separate panels for each genotype. Mean GFP fluorescence intensity—corrected by subtracting local background fluorescence then by subtracting the minimum value for the entire experiment, to avoid negative values (see Methods, vertical axis)—is plotted against microcolony growth rate for (A) FY4 no-GFP control (black, 3915 microcolonies), (B) *TSL1-GFP* (green, 10531 microcolonies), (C) *TSL1-GFP msn2* (light purple, 6460 microcolonies), (D) *TSL1-GFP msn4* (light orange, 3724 microcolonies), and (E) *TSL1-GFP msn2 msn4* (light blue, 5621 microcolonies). Each solid line is the fit to a generalized additive model with cubic spline smoother, with 95% confidence interval shown in yellow. Vertical axis is on a square-root scale for a better view at the low-intensity end.
(TIF)

S5 Fig Unexpected effects of PKA mutants on growth-rate heterogeneity. Growth-rate cumulative density curves of FY4 (black, 5589 microcolonies), *tpk1* (orange, 8556 microcolonies), *tpk2* (blue, 7282 microcolonies) and *bcy1* (yellow, 1146 microcolonies). Vertical axis is on a square-root scale for a better view of the slower-growing tail of each distribution.
(TIF)

S6 Fig Treatment with PKA inhibitor H89 increases Msn2 nuclear occupancy. Cumulative density plot of relative Msn2 nuclear abundance for FY4 without H89 treatment (solid, black line, 2399 cells) or treated with 75 μM H89 (solid, red line, 2190 cells). The matched DMSO-only control (2339 cells) is shown as the dashed, red line.
(TIF)

S1 File Cell Profiler project for cell and nucleus recognition. (CPPROJ)

S2 File Msn2 subcellular localization with H89 treatment. (CSV)

S3 File Time series of Msn2 subcellular localization with subsequent microcolony growth rate under benign conditions. (CSV)
