# Supplementary figures and images for: Control of nongenetic heterogeneity in growth rate and stress tolerance of Saccharomyces cerevisiae by cyclic AMP-regulated transcription factors
Source: PLoS Genet. 2018 Nov 2;14(11):e1007744. doi: 10.1371/journal.pgen.1007744 (PMC6241136; doi:10.1371/journal.pgen.1007744)

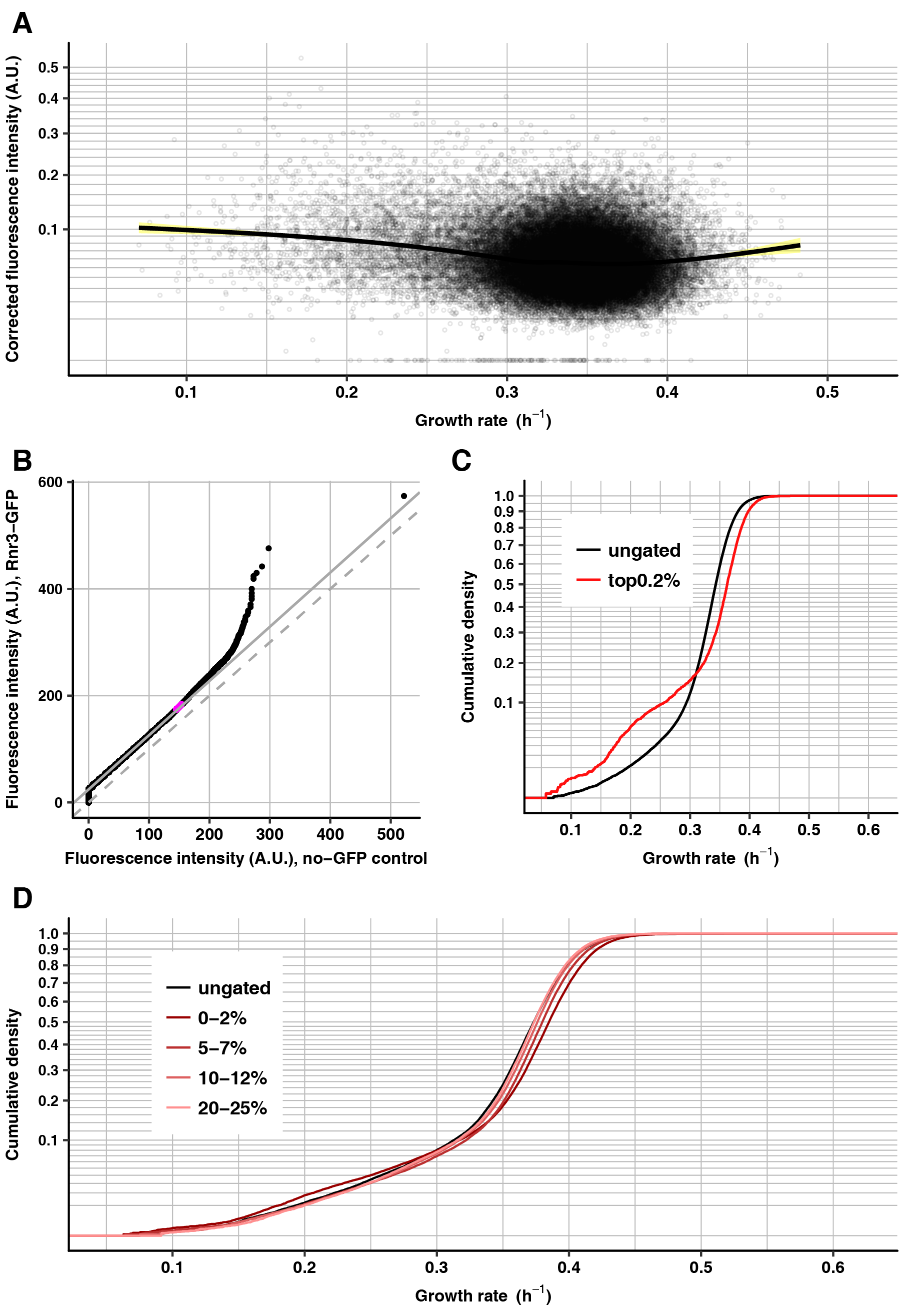

Supplement: S1 Fig — (A) Mean Hsp12-mCherry fluorescence intensity—corrected by subtracting local background fluorescence then by subtracting the minimum value for the entire experiment, to avoid negative values (see Methods, vertical axis)—is plotted against microcolony growth rate (horizontal axis). Total sample size is 59183 microcolonies. The solid line is the fit to a generalized additive model with cubic spline smoother, with 95% confidence interval shown in yellow. Vertical axis is on a square-root scale for a better view at the low-intensity end. (B) Quantile-quantile plot of fluorescence intensity of Rnr3-GFP strain versus no-GFP control. For each strain 12978 cells were recorded. The dashed grey line has intercept 0 and slope 1; the solid grey line is the least-squares linear best fit of the quantile-quantile plot between the bottom 10% and 25% quantiles. Points highlighted in magenta correspond to the region between the top 20% and 25% quantiles. (C) Growth-rate cumulative density curves of FACS-gated top 0.2% RNR3-GFP cells (red, 4556 microcolonies) and ungated cells (black, 59183 microcolonies). Vertical axis is on a square-root scale for a better view of the slower-growing tail of each distribution. (D) Growth-rate cumulative density curves of the following FACS-gated bins of RNR3-GFP cells with 0% being the most intense: 0–2% (43393 microcolonies), 5–7% (44201 microcolonies), 10–12% (41465 microcolonies), 20–25% (37048 microcolonies) (shown in increasingly light shades of red), and ungated cells (black, 39617 microcolonies). Vertical axis is on a square-root scale for a better view of the slower-growing tail of each distribution. (TIF) [file pgen.1007744.s001.tif]

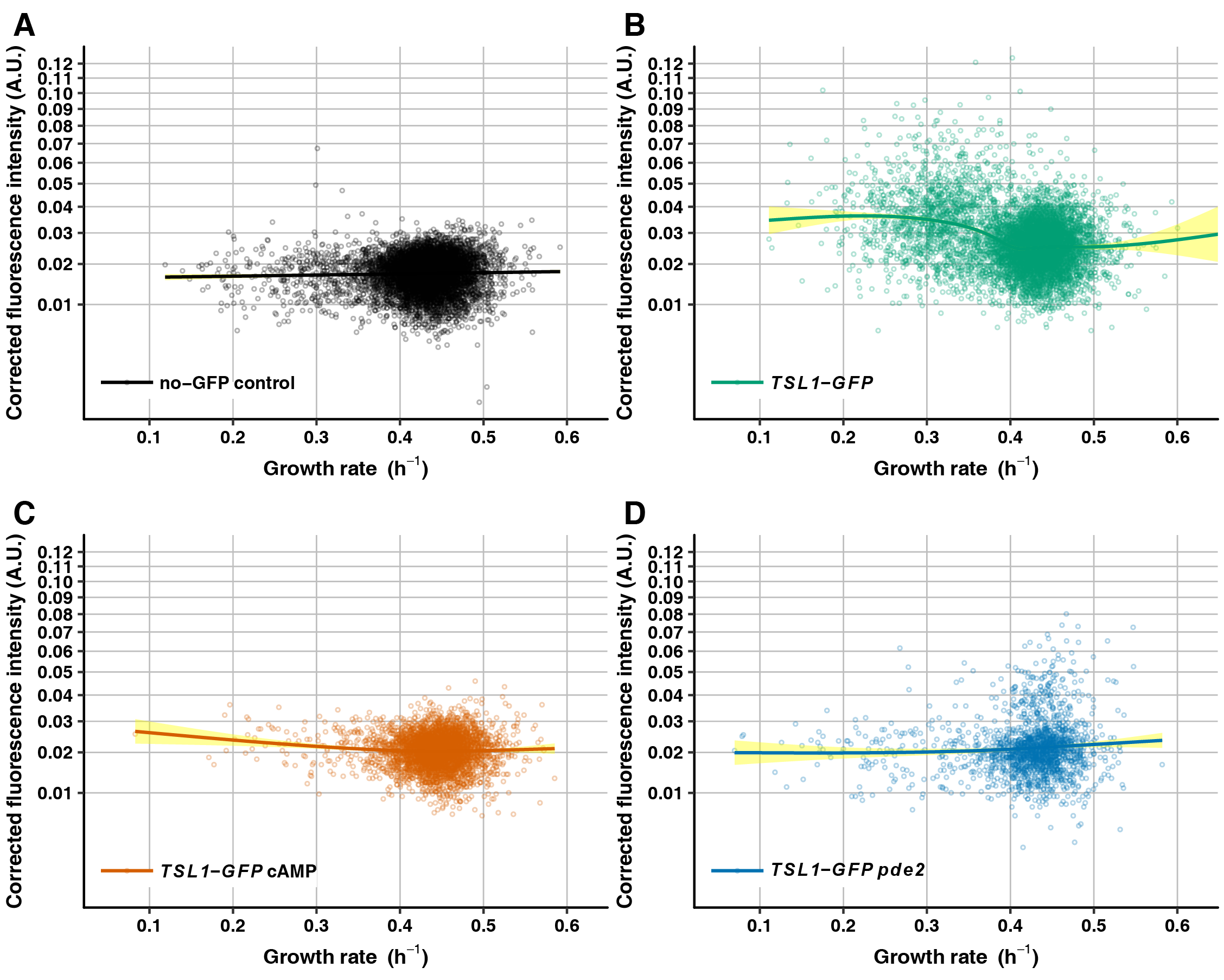

Supplement: S2 Fig — Same data as in Fig 4B plotted in separate panels for each genotype or treatment. Mean GFP fluorescence intensity—corrected by subtracting local background fluorescence then by subtracting the minimum value for the entire experiment, to avoid negative values (see Methods, vertical axis)—is plotted against microcolony growth rate (horizontal axis) for (A) FY4 no-GFP control (black, 7340 microcolonies), (B) TSL1-GFP (green, 6912 microcolonies), (C) TSL1-GFP cultivated with 15 mM 8-bromo-cAMP (orange, 3730 microcolonies) and (D) TSL1-GFP pde2 (blue, 1778 microcolonies). Each solid line is the fit to a generalized additive model with cubic spline smoother, with 95% confidence interval shown in yellow. Vertical axis is on a square-root scale for a better view at the low-intensity end. (TIF) [file pgen.1007744.s002.tif]

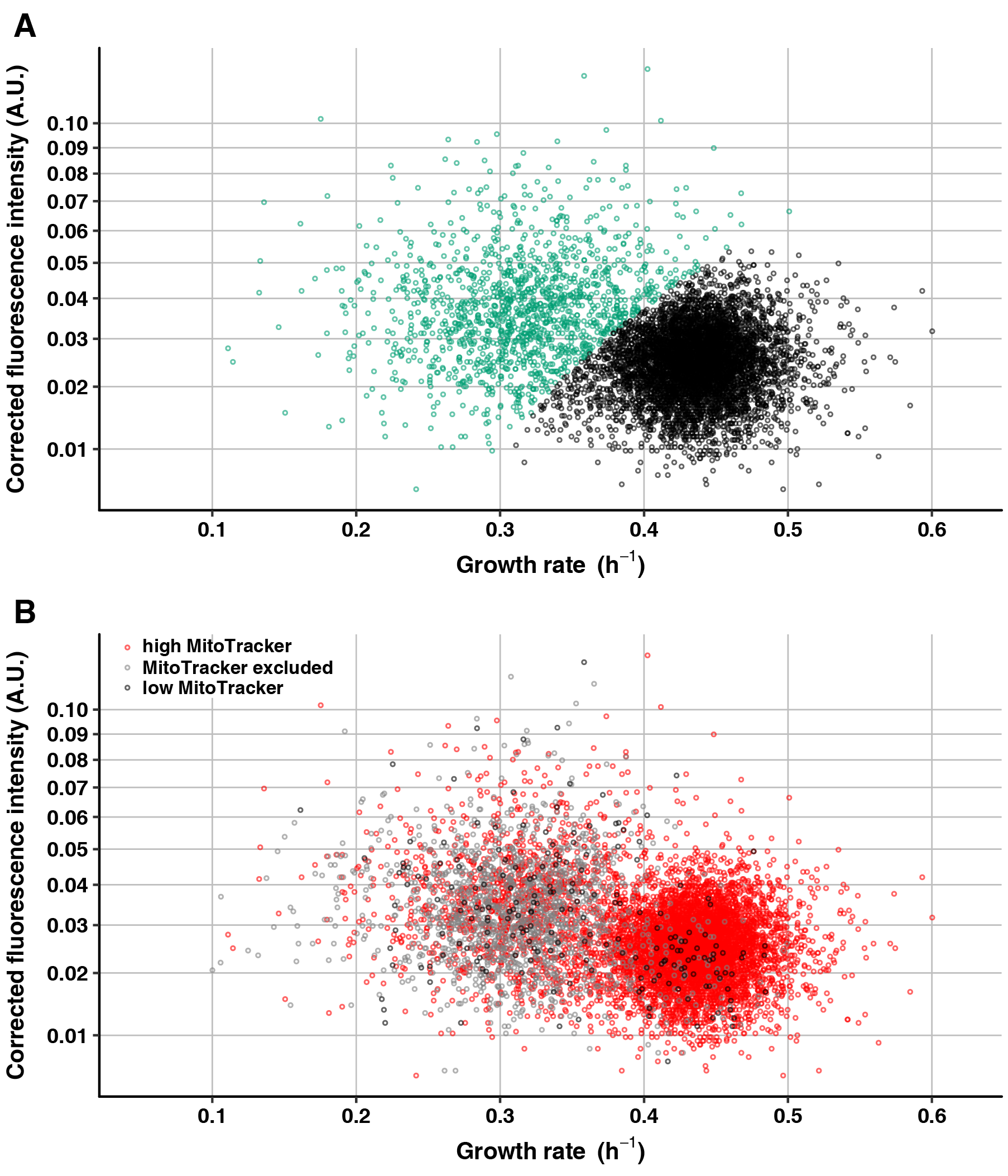

Supplement: S3 Fig — (A) Same plot of TSL1-GFP data as in Fig 4B, with two clusters identified by partitioning around medoids indicated in black (higher growth rate, lower Tsl1 abundance) and green (lower growth rate, higher Tsl1 abundance). (B) Same plot of TSL1-GFP data as in Fig 4B, with microcolonies color coded by MitoTracker staining (black = lowest 3% of MitoTracker staining of microcolonies that passed the MitoTracker-staining threshold, red = highest 97% of microcolonies that passed the MitoTracker staining) and with additional data shown for microcolonies that had not passed the MitoTracker-staining threshold (grey). (TIF) [file pgen.1007744.s003.tif]

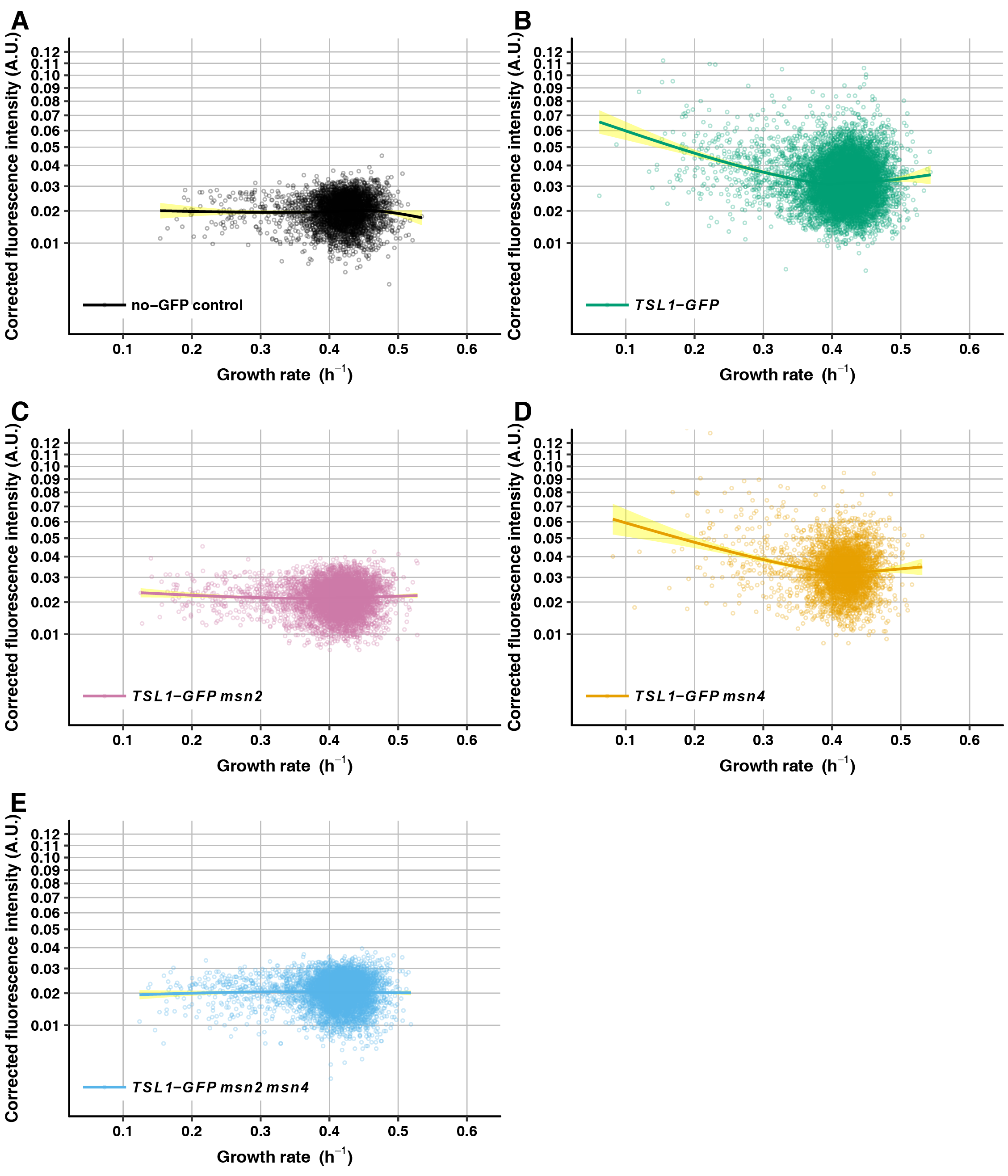

Supplement: S4 Fig — Same data as in Fig 6B plotted in separate panels for each genotype. Mean GFP fluorescence intensity—corrected by subtracting local background fluorescence then by subtracting the minimum value for the entire experiment, to avoid negative values (see Methods, vertical axis)—is plotted against microcolony growth rate for (A) FY4 no-GFP control (black, 3915 microcolonies), (B) TSL1-GFP (green, 10531 microcolonies), (C) TSL1-GFP msn2 (light purple, 6460 microcolonies), (D) TSL1-GFP msn4 (light orange, 3724 microcolonies), and (E) TSL1-GFP msn2 msn4 (light blue, 5621 microcolonies). Each solid line is the fit to a generalized additive model with cubic spline smoother, with 95% confidence interval shown in yellow. Vertical axis is on a square-root scale for a better view at the low-intensity end. (TIF) [file pgen.1007744.s004.tif]

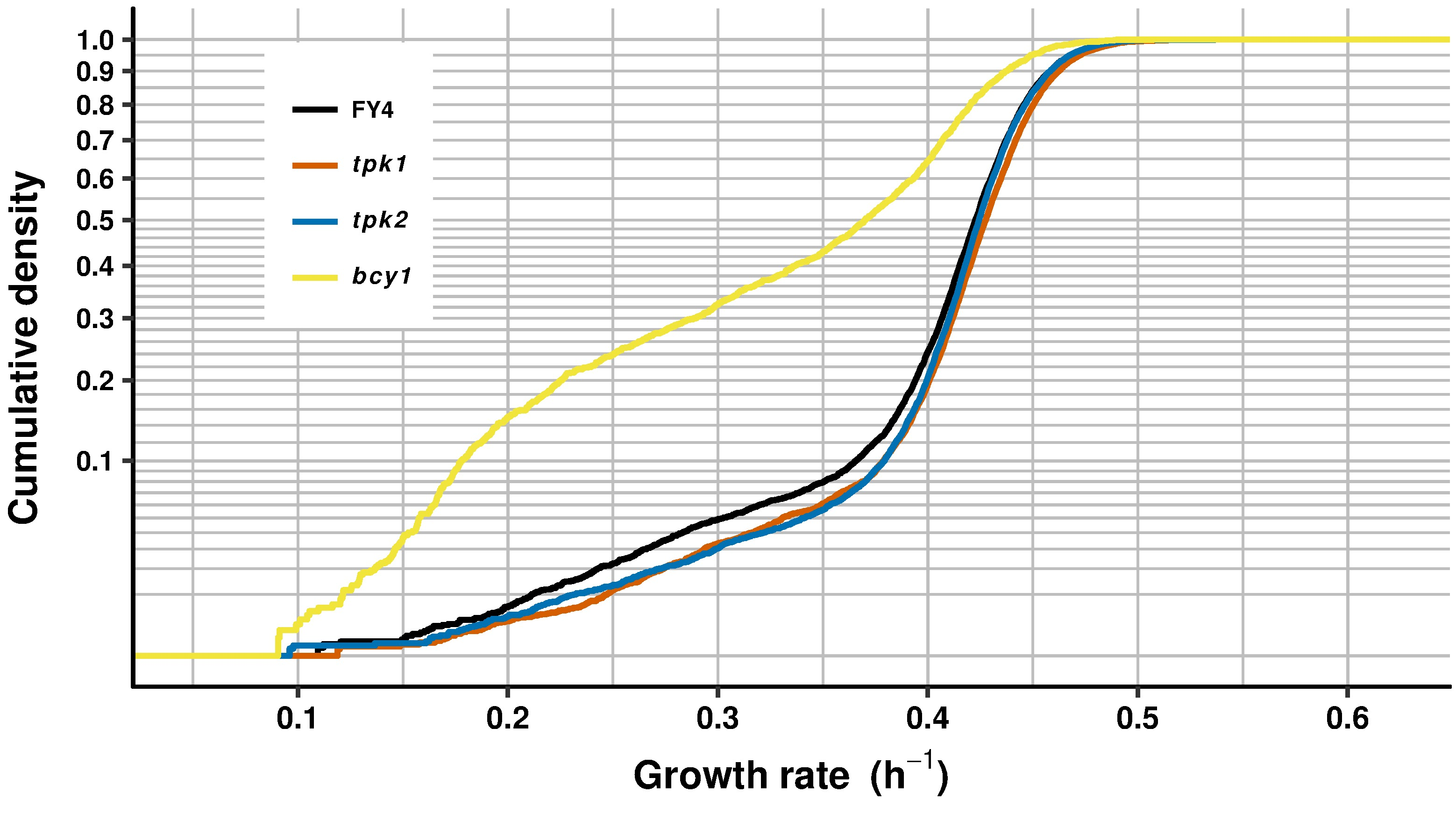

Supplement: S5 Fig — Growth-rate cumulative density curves of FY4 (black, 5589 microcolonies), tpk1 (orange, 8556 microcolonies), tpk2 (blue, 7282 microcolonies) and bcy1 (yellow, 1146 microcolonies). Vertical axis is on a square-root scale for a better view of the slower-growing tail of each distribution. (TIF) [file pgen.1007744.s005.tif]

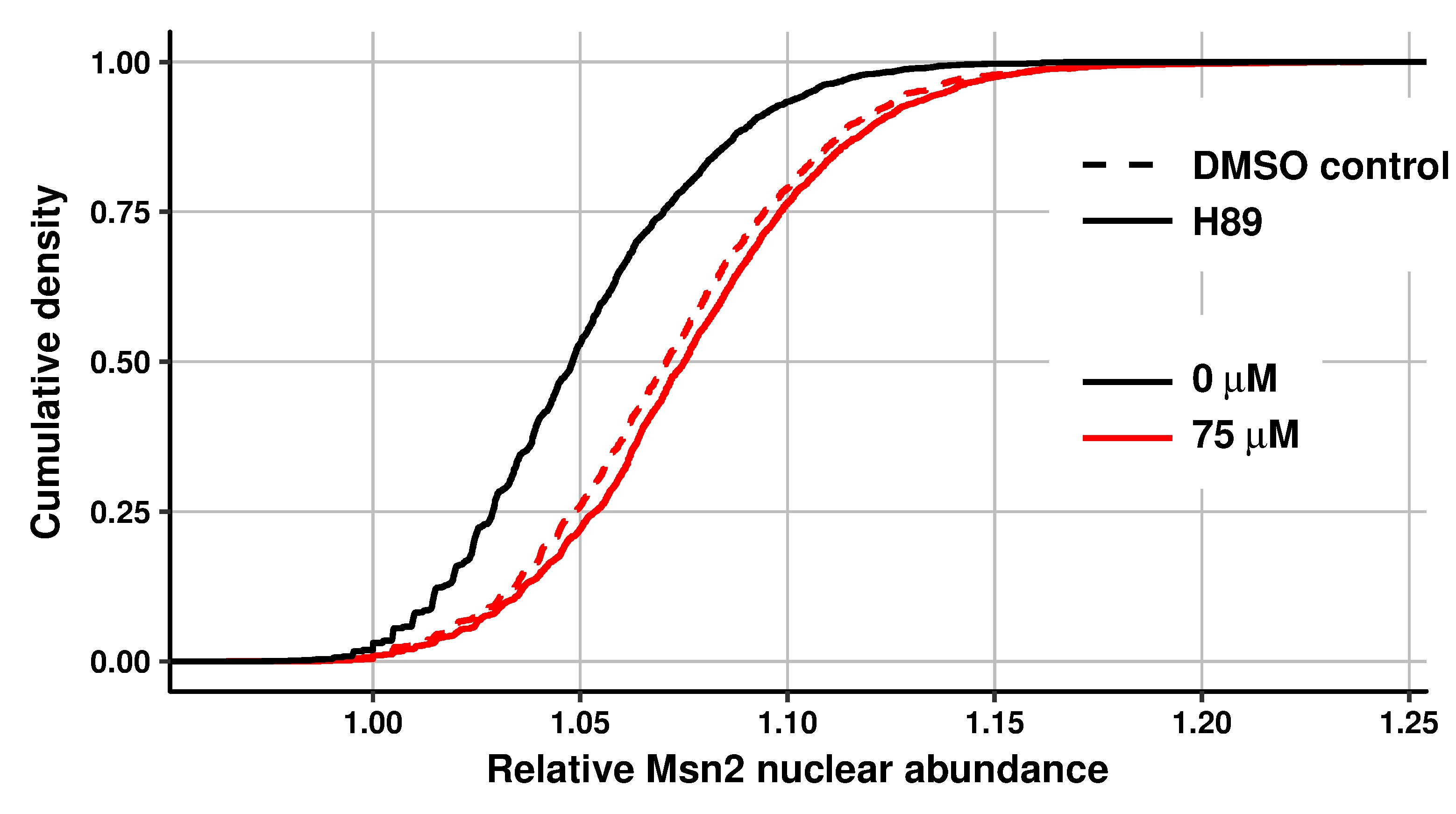

Supplement: S6 Fig — Cumulative density plot of relative Msn2 nuclear abundance for FY4 without H89 treatment (solid, black line, 2399 cells) or treated with 75 μM H89 (solid, red line, 2190 cells). The matched DMSO-only control (2339 cells) is shown as the dashed, red line. (TIF) [file pgen.1007744.s006.tif]
